# Target-Dependent Coordinated Biogenesis of Secondary MicroRNAs by miR-146a Balances Macrophage Activation Processes

**DOI:** 10.1128/mcb.00452-21

**Published:** 2022-03-21

**Authors:** Susanta Chatterjee, Ishita Mukherjee, Shreya Bhattacharjee, Mainak Bose, Saikat Chakrabarti, Suvendra N. Bhattacharyya

**Affiliations:** a RNA Biology Research Laboratory, Molecular Genetics Division, CSIR-Indian Institute of Chemical Biology, Kolkata, West Bengal, India; b Structural Biology and Bioinformatics Division, CSIR-Indian Institute of Chemical Biology, Kolkata, West Bengal, India; c Academy of Scientific and Innovative Research (AcSIR), CSIR-Human Resource Development Centre, Ghaziabad, Uttar Pradesh, India

**Keywords:** coordinated biogenesis of miRNAs, anti-inflammatory miRNAs, macrophage polarization, target-dependent miRNA biogenesis, activated macrophages, cooperative miRNA biogenesis, macrophage activation, primary miRNA, target mRNA-dependent miRNA biogenesis, miRNA biogenesis

## Abstract

MicroRNAs (miRNAs) repress protein expression by binding to the target mRNAs. Exploring whether the expression of one miRNA can regulate the abundance and activity of other miRNAs, we noted the coordinated biogenesis of miRNAs in activated macrophages. miRNAs with higher numbers of binding sites (the “primary” miRNAs) induce expression of other miRNAs (“secondary” miRNAs) having binding sites on the 3′ untranslated region (UTR) of common target mRNAs. miR-146a-5p, in activated macrophages, acts as a “primary” miRNA that coordinates biogenesis of “secondary” miR-125b, miR-21, or miR-142-3p to target new sets of mRNAs to balance the immune responses. During coordinated biogenesis, primary miRNA drives the biogenesis of secondary miRNA in a target mRNA- and Dicer1 activity-dependent manner. The coordinated biogenesis of miRNAs was observed across different cell types. The target-dependent coordinated miRNA biogenesis also ensures a cumulative mode of action of primary and secondary miRNAs on the secondary target mRNAs. Interestingly, using the “primary” miR-146a-5p-specific inhibitor, we could inhibit the target-dependent biogenesis of secondary miRNAs that can stop the miRNA-mediated buffering of cytokine expression and inflammatory response occurring in activated macrophages. Computational analysis suggests the prevalence of coordinated biogenesis of miRNAs also in other contexts in human and in mouse.

## INTRODUCTION

MicroRNAs (miRNAs) are small regulatory RNAs that are primarily processed from introns, exons, or intergenic regions of the mammalian genome by the canonical or noncanonical pathway of miRNA biogenesis (1). After sequential processing, the precursor form of the miRNA is cleaved by the endonuclease Dicer1 to form the miRNA/miRNA* (guide and passenger miRNA strands) duplex and the miRNA-encoding strand gets loaded onto Argonaute proteins to form the RNA-induced silencing complex (miRISC) for silencing of target mRNAs (2). Conventionally, miRNA binds to the target mRNA with 5′ conserved seed sequence complementarity and represses the expression of proteins by translational repression and/or destabilization of target mRNA.

Intracellular levels of different miRNAs produced during development in specific cell types or in response to specific environmental cues may be governed by transcription factors, posttranscriptional regulatory mechanisms influencing miRNA processing, or abundance of precursor miRNA (3, 4). In a reciprocal fashion, miRNA activity and abundance can be altered by multiple factors, including RNAs which themselves are under miRNA regulation. A group of coding and non-coding RNAs (pseudogenes, long non-coding RNAs [lncRNAs], circular RNA [circRNA]) can modulate the miRNA activity by forming an integrative network (5). This interconnected regulatory cross talk gives rise to the idea of the competitive endogenous RNA (ceRNA) hypothesis, where ceRNA with miRNA binding sites (also referred as miRNA response elements [MRE]) compete for miRNA binding against the endogenous target mRNAs that ultimately lead to derepression of the target mRNA through the “miRNA sponge effect” of ceRNA (6). A well-established network of this mode of regulation is that of PTEN mRNA, a critical tumor suppressor-encoding mRNA regulated by various group of ceRNAs (7, 8). The functional sequestration of miRNA-122 (miR-122) in hepatic cells by a hepatitis C virus (HCV) synthetic construct also manifests the “sponge effect” to elicit functional derepression of the miR-122 targetome (9). Although ceRNA-mediated derepression could be exacerbated by overexpression of different synthetic RNA transcripts, the concept is debatable due to the lack of abundant physiological levels of ceRNAs in a cellular context (10).

There is ample evidence for different modes of regulatory control of RNA over miRNA biogenesis and activity. Kleaveland et al. have shown a non-coding RNA (ncRNA) network where different species of ncRNA cooperatively act to modulate neuronal activity in the brain (11). It has also been reported how an increase in the number of binding sites on a target mRNA increases the “processivity” of Dicer1, leading to enhanced cognate miRNA biogenesis from a precursor in hepatocytes subjected to refeeding after amino acid starvation (12). We were interested to dissect the consequent effect of this regulation by target mRNA of a specific miRNA on other miRNAs that are sharing *cis*-binding sites on the same target mRNAs’ 3′ untranslated region (UTR). Does there exist a cooperative biogenesis of miRNAs that share binding sites on the 3′ UTR of common target mRNAs? Or, in contrast, is there any competitive binding among groups of miRNAs on the 3′ UTR of common target mRNAs? The answers are not yet known.

To address this question, we wanted to explore a physiologically relevant system where cross talk of multiple miRNAs may fine-tune common signaling pathways. TLR4-activated inflammation, elicited by exogenous or endogenous ligands, does give rise to several acute and chronic diseases and plays a critical role as an amplifier of the inflammatory responses. Expression profiling in human monocytes revealed groups of endotoxin-responsive miRNAs which fine-tune the expression of signaling mediators during inflammatory escalation (13, 14). miRNAs can stimulate or regulate various signaling pathways such as the TLR‐signaling pathways (TLR4, TLR3, and TLR7/8, etc.), the NF‐Kβ pathway, or MAPK pathways (MAPK/ERK, MAPK/JNK, and MAPK/p38 pathways) to mount pro‐ and anti‐inflammatory responses to modulate innate immune responses (15, 16). We anticipated the existence of molecular coordination among those miRNAs teaming up to regulate the immune responses. We hypothesize that the coordination might be achieved by target mRNAs affecting the biogenesis of multiple miRNAs to create a regulatory module in infected macrophages.

We have tested and validated our hypothesis of coordinated miRNA biogenesis in mammalian cells. Adopting the endotoxin-activated macrophage system, we documented the cooperative biogenesis of miRNAs. We observed that miR-146a-5p could modulate the biogenesis and activities of groups of miRNAs like miR-125b, miR-142-3p, and miR-21. In turn, this phenomenon resulted in repression of secondary target mRNAs that are controlled by the newly generated secondary miRNAs. These network-based regulations of miRNAs allowed us to propose a target-dependent cooperative biogenesis (TDCB) in mammalian cells, where miRNA species with higher numbers of binding sites influence the biogenesis and activity of a group of “cooperative” miRNAs having target sites on the same common mRNAs. Considering this observation, a comprehensive computational analysis has also been performed to find the general prevalence of the coordinated biogenesis (CB) phenomenon for multiple miRNAs in both human and mouse. Our observations also suggest that this “epistatic” mode of regulation of miRNA can in turn influence and fine-tune different cellular signaling pathways essential for reimposition of cellular homeostasis under a changed environment.

## RESULTS

### Stepwise expression of miRNAs in activated macrophages.

**FIG 1:**
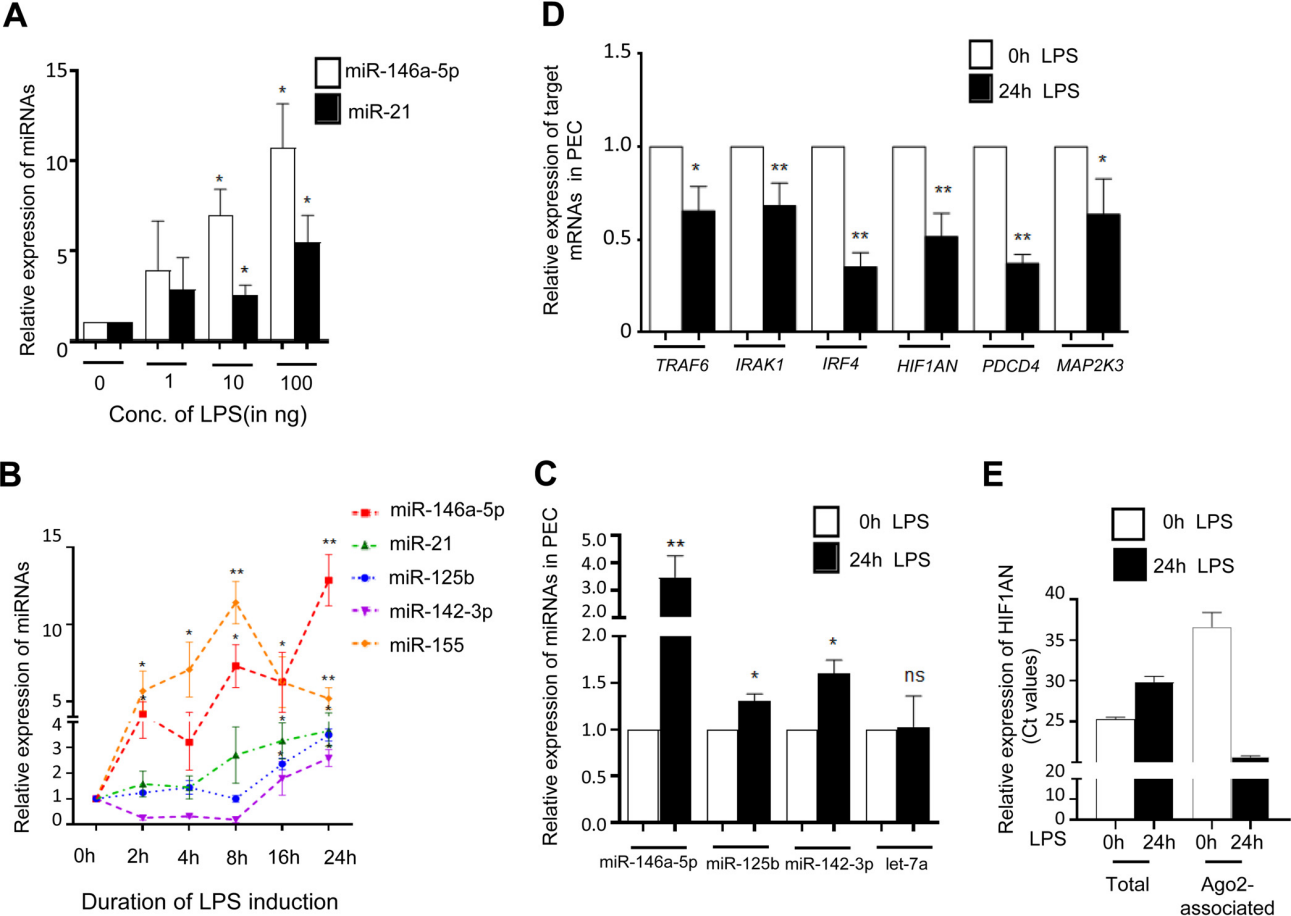
LPS induces expression of groups of miRNAs in murine macrophages. (A) Expression of miR-146a-5p and miR-21 in murine macrophages after treatment with increasing concentrations of LPS. PCR data reveals a dose-dependent increase of miR-146a-5p and miR-21 expression levels after LPS treatment for 24 h in RAW264.7 cells (*n* = 3). (B) Changes in the levels of different miRNAs against time during LPS (10 ng/mL) treatment. qRT-PCR data show changes in miR-155, miR-146a-5p, miR-125b, miR-142-3p, and miR-21 expression after LPS induction. Values at 0 h are taken as units (*n* = 3). (C to E) LPS treatment of PEC increases the expression of LPS-responsive (miR-146a-5p, miR-125b, miR-142-3p) but not nonresponsive (let-7a) miRNAs. (C) Treatment was done with 10 ng/mL of LPS for 24 h. (D) Real-time quantification of respective targets. TRAF6 and IRAK1 (of miR-146a), IRF4 and HIF1AN (of miR-125b), and PDCD4 and MAP2K3 (of miR-21) were measured and plotted after 24 h of LPS treatment. (E) Relative levels of total and Ago2-associated HIF1AN mRNA in control versus 24-h LPS-treated PEC. Average *C_T_* values are plotted for each sample (*n* = 3). 18s rRNA or GAPDH and U6 snRNA were used as endogenous targets in qRT-PCR of mRNA and miRNA quantification, respectively. Student’s *t* tests were used for all comparisons. *P* < 0.05 (*); *P* < 0.01 (**); *P* < 0.001 (***); *P* < 0.0001 (****).

Bacterial membrane lipopolysaccharide (LPS) is an endotoxin synthesized by Gram-negative bacteria that serves as an immediate activator of the TLR4 signaling pathway to increase the production of proinflammatory cytokines which protect host cells by the innate immune response (17). In activated macrophages, the miRNA activity loss due to miRNA uncoupling from Ago proteins precedes the de *novo* miRNA biogenesis and reestablishment of the miRNA-mediated repression of target cytokine mRNAs during prolonged exposure to LPS (18, 19). In this process, the reassociation of Ago2 with housekeeping miRNAs has been observed (i.e., let-7a miRNA) while increased expression of several new miRNAs has also been noted (14, 20). Interestingly, a majority of these miRNAs are induced in a phase-wise manner, and the transcriptional surge of the precursors encoding these miRNAs has not been sufficient to explain the cascaded expression pattern observed for these miRNAs in mammalian macrophages. A group of miRNAs are proven to be the key mediators of TLR4-responsive pathways, and among them, miR-21 and miR-146a-5p show a dose-dependent increase after LPS-induced activation of murine macrophages (Fig. 1A) (21). These miRNAs also follow a specific temporal expression pattern after LPS exposure. While miR-155 shows a peak of expression at an early time point, miR-146a-5p and miR-21 are induced at comparably later time points (Fig. 1B) (13, 22, 23). miR-125b and miR-142-3p are also induced at later time points (Fig. 1B) (20, 24, 25). An increase in specific miRNAs in LPS-treated primary macrophages or peritoneal exudate cells (PEC) treated with LPS has also been documented (Fig. 1C). The induced expression of these miRNAs is associated with reduced expression but increased Ago2 association of the miR-125b target mRNA *HIF1AN* in macrophages after 24 h of LPS exposure, confirming the functionally active forms of the induced miRNAs (Fig. 1D and E).

### miR-146a-5p coordinates biogenesis of groups of secondary miRNAs.

**FIG 2:**
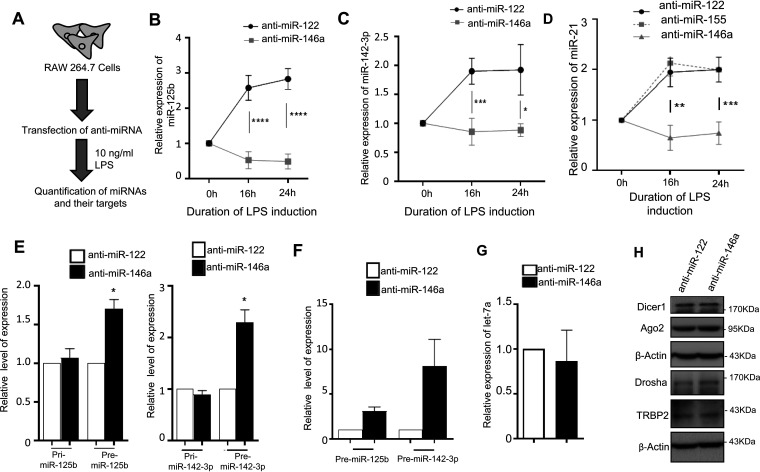
miR-146a-5p regulates biogenesis of groups of miRNAs in LPS-induced murine macrophages. (A) Schematic representation of experiments done with miR-146a-5p-inactivated macrophages treated with LPS. RAW264.7 murine macrophages were transfected with either 30 nM anti-miR-146a or anti-miR-122 (control) oligonucleotides. After transfection, cells were induced with 10 ng/mL LPS for the respective times. (B and C) miR-125b (B) and miR-142-3p (C) levels after LPS induction in anti-miR-146a- or anti-miR-122-transfected RAW264.7 murine macrophages. Relative levels of miRNA were estimated by qRT-PCR and plotted (*n* = 3). Values at 0 h are taken as units. (D) Cellular levels of miR-21 in anti-miR-146a-, anti-miR-122-, and anti-miR-155-transfected RAW264.7 cells after LPS treatment. Relative levels of miRNA were estimated by qRT-PCR and plotted (*n* = 2). (E) Relative levels of primary (pri-) and precursors (pre-) of miR-125b or miR-142-3p in anti-miR-146a-transfected RAW264.7 cells treated with LPS for 24 h (*n* = 3). Values in control anti-miR-122-transfected cells were set as units. (F) Cellular levels of pre-miR-125b and pre-miR-142-3p in anti-miR-146a-transfected RAW264.7 cells treated with LPS for 24 h (*n* = 3). Values in control anti-miR-122-transfected cells were set as units. The small-RNA population isolated with the small-RNA-specific mirVana RNA isolation kit was used for the analysis (*n* = 3). (G) Relative level of let-7a in anti-miR-146a-transfected LPS-treated RAW264.7 cells (*n* = 3). Values in control anti-miR-122-transfected cells were set as units. (H) Cellular levels of miRNP-associated proteins in anti-miR-146a- and control anti-miR-122-transfected LPS-treated macrophages. miRNP-associated proteins and processing enzymes Dicer1, Ago2, TRBP2, and Drosha were Western blotted in miR-146a-5p-inactivated LPS-treated RAW264.7 cells. β-Actin served as an endogenous control. 18s rRNA or GAPDH and U6 snRNA were used as endogenous targets in qRT-PCR of mRNA and miRNA quantification, respectively. Student’s *t* tests were used for all comparisons. *P* < 0.05 (*); *P* < 0.01 (**); *P* < 0.001 (***); *P* < 0.0001 (****).

A notion that the late expression of certain miRNAs upon LPS activation is coupled with the expression of specific miRNAs which are induced early during LPS treatment has prompted us to test the effect of miR-146a-5p inhibition on expression of miR-125b, miR-142-3p, and miR-21. We observed a significant decrease in miR-125b and miR-142-3p levels in LPS-treated macrophages inhibited for miR-146a-5p using anti-miR-146a antagomir but not in control anti-miR-122 (a liver-specific miRNA not expressed in macrophages)-treated cells (Fig. 2A to C). Anti-miR-122 treatment has no effect on cellular miRNA levels or activity in comparison to control nonspecific antagomir treatment (data not shown) and therefore has been used throughout this study as a control. In contrast, precursors of both the miRNAs showed increased accumulation, whereas no significant change in the levels of respective primary miRNAs were noted (Fig. 2E and F). The data correlate with unchanged levels of transcription but decreased processing of miR-125b or miR-142-3p from their respective precursors in anti-miR-146a-treated cells. We could also observe a similar trend for mature miR-21 in anti-miR-146a-treated cells while miR-21 levels remained unchanged after treatment with either control anti-miR-122 or anti-miR-155 (a proinflammatory miRNA expressed at an early time of LPS activation), used as another control (Fig. 2D). let-7a expression, a non-cooperative miRNA, remained unaltered post-LPS induction (18), and the level of let-7a did not change even after miR-146a-5p inhibition (Fig. 2G). These observations reflect the specific regulatory role of miR-146a-5p, as a primary miRNA, on the biogenesis of specific secondary miRNAs such as miR-142-3p, miR-125b, or miR-21 in LPS-stimulated macrophages. To understand the molecular events that control the miR-146a-5p-dependent regulation of these late induced miRNAs in macrophages, we followed the expression levels of different miRNA-associated protein factors, namely, Dicer1, Ago2, and TRBP2, which are known to be associated with processing of primary (pri-) and precursor (pre-)miRNAs. We could not observe any change in expression of protein factors associated with miRNA biogenesis, negating the possibilities of miR-146a-5p-mediated upregulation of a pan-miRNP processing event that can account for the generic miRNA level change in activated macrophages (Fig. 2H).

### Presence of functional secondary miRNA binding sites on mRNAs having target sites for primary miRNA-146a-5p.

**FIG 3:**
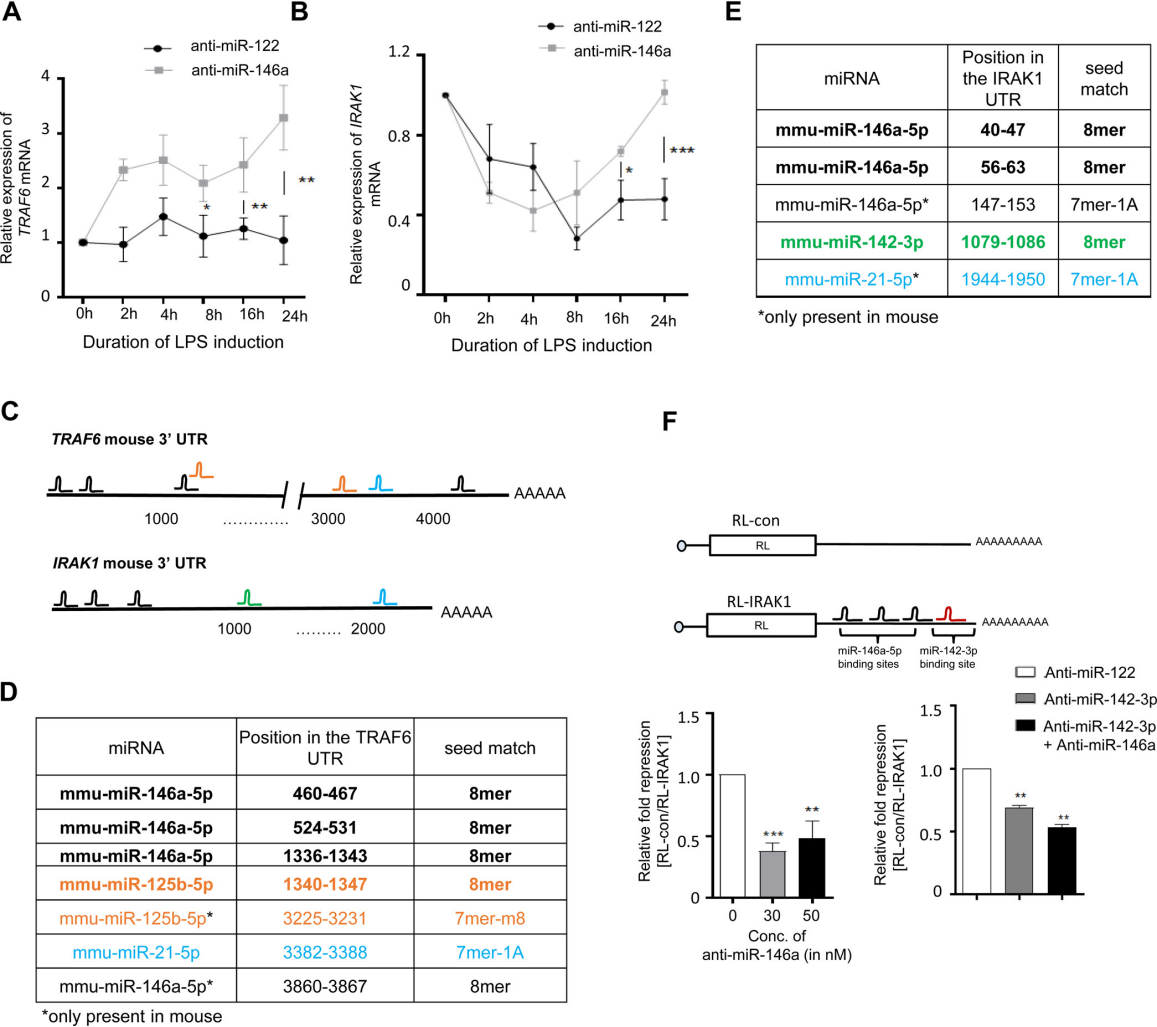
miR-146a-5p and coregulated secondary miRNAs share binding sites on different mRNAs encoding TLR4 signaling components. (A and B) Expression of TRAF6 (A) and IRAK1 (B) mRNAs in anti-miR-146a- and control anti-miR-122-transfected macrophages after 10 ng/mL of LPS treatment over 0- to 24-h time points (*n* = 3). Relative levels of miRNA were estimated by qRT-PCR and plotted (*n* = 3). Values at 0 h are taken as units. (C to E) Schematic representation of the 3′ UTRs of TRAF6 and IRAK1 mRNA. (C) The respective miRNA binding sites are shown on the 3′ UTR of these two mRNAs. TRAF6 and IRAK1 murine mRNAs both bear single and nonconserved miR-21 binding sites on their 3′ UTR as predicted by TargetScan. (D and E) The exact positions of the miRNA binding sites and the types of seed matches of binding sites are listed. (F) A schematic representation of reporter RL-IRAK1 mRNA with 3′ UTR of IRAK1 mRNA with respective miRNA binding sites is shown in the upper panel. Relative levels of *Renilla* luciferase reporter RL-IRAK1 expression in cells transfected with increasing concentrations of anti-miR-146a are shown in the left panel. Relative repression of the RL-IRAK1 reporter in cells transfected without anti-miR-146a oligonucleotides has been taken as units. Relative levels of the *Renilla* luciferase reporter RL-IRAK1 in anti-miR-146a-transfected or both anti-miR-142-3p- and anti-miR-146a-transfected LPS-treated RAW264.7 cells are shown in the right panel. RL-con or RL-IRAK1 reporters were cotransfected with firefly luciferase (FF) expression plasmid, where FF expression was used as a transfection control (*n* = 3). Relative repression levels of RL-IRAK1 reporter in control anti-miR-122-transfected cells were taken as units. 18s rRNA or GAPDH and U6 snRNA were used as endogenous targets in qRT-PCR of mRNA and miRNA quantification, respectively. Student’s *t* tests were used for all comparisons. *P* < 0.05 (*); *P* < 0.01 (**); *P* < 0.001 (***); *P* < 0.0001 (****).

miR-146a-5p is a critical immune regulator and regulates Toll-like receptor and cytokine signaling via a negative-feedback regulation loop mediated through downregulation of interleukin 1 (IL-1) receptor-associated kinase 1 (IRAK1) and tumor necrosis factor (TNF) receptor-associated factor 6 (TRAF6) protein levels (13). TLR4 signaling components TRAF6 or IRAK1 encoding mRNAs bearing multiple miR-146a-5p binding sites showed synchronized expression with miR-146a-5p in a time-dependent manner and showed derepression after miR-146a-5p-specific inhibition in LPS-induced macrophages (Fig. 3A and B) (26).

Different predicted (TargetScan) and experimentally validated (miRTarBase) databases and previous publications based on combinatorial analysis of TLR4 pathway-miRNA network study revealed that TRAF6 bears one conserved miR-125b binding site and IRAK1 possesses one conserved miR-142-3p binding site along with previously mentioned miR-146a-5p binding sites which are present on both targets. These sites are conserved across mammals in the phylogenetic tree (Fig. 3C to E) (25, 27, 28). miR-21 is another critical modulator of LPS-responsive pathways (23), and TargetScan and previous reports also reveal that the 3′ UTRs of both TRAF6 and IRAK1 contain single miR-21-5p binding sites along with multiple miR-146a-5p sites in mouse genes (Fig. 3C to E) (27, 29). Possession of active binding sites for both miR-142-3p and miR-146a-5p on a *Renilla* luciferase (RL) reporter with IRAK1 3' UTR (RL-IRAK1-3′ UTR) has been reconfirmed using a luciferase assay with respective anti-miR inhibitors (Fig. 3F). Therefore, it is possible that the common mRNA targets may play a role in induction of secondary miRNAs in the presence of primary miRNA. The primary miRNA level may thus influence the biogenesis and activity of a group of secondary miRNAs within a cooperative group of miRNAs which are under primary miRNA control.

### Target-driven coordinated biogenesis of secondary miRNAs is coupled with cooperative repression of their targets in murine macrophages.

**FIG 4:**
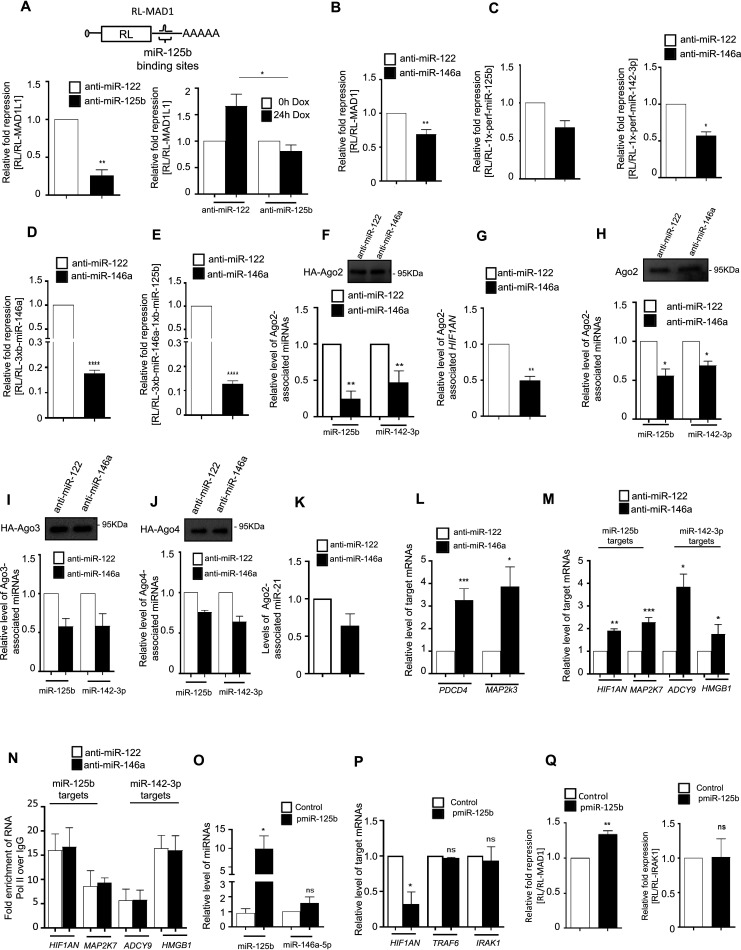
Coordinated biogenesis of secondary miRNAs driven by miR-146a-5p is linked with increased activity of secondary miRNAs in activated macrophages. (A) Schematic representation of the RL-MAD1 luciferase reporter mRNA with one miR-125b binding site on its 3′ UTR. A dual-luciferase assay shows reduction in repressive activity of miR-125b in anti-miR-125b-transfected RAW264.7 cells treated with LPS (left panel). Repression of RL-MAD1 was observed in LPS-treated anti-miR-125b-treated cells after 24 h of miR-146a induction (right panel). Repression levels at 0 h for both anti-miR-transfected cells are taken as units. (B) A dual-luciferase assay shows reduction in miR-125b activity in anti-miR-146a-transfected RAW264.7 cells treated with LPS. A *Renilla* reporter without any miRNA binding sites (RL-con) was used as a control to get the relative level of repression represented as the ratio of FF normalized RL-con to RL-MAD1 expression. A firefly luciferase (FF) construct without miRNA sites was used to normalize the transfection efficiency between the sets used for this assay (*n* = 3). (C) A dual-luciferase assay was used to score the level of repression for miR-125b or miR-142-3p reporters (RL-1x-perf-miR-125b and RL-1x-perf-miR-142-3p, respectively) in control anti-miR-122- or anti-miR-146a-transfected RAW264.7 cells treated with LPS. A *Renilla* reporter without any miRNA binding sites (RL-con) was used as a control. An FF construct without miRNA sites was used to normalize the transfection efficiency between the sets (*n* = 3). Values obtained with anti-miR-122-transfected cells were taken as units. (D and E) A dual-luciferase assay shows a reduced level of repression for miR-146a-5p reporter alone (RL-3xb-miR-146a) (D) and along with dual miR-146a/miR-125b reporter having binding sites for both miRNAs (RL-3xb-miR-146a-1xb-miR-125b) (E) in control anti-miR-122- and anti-miR-146a-transfected RAW264.7 cells treated with LPS. A *Renilla* reporter without any miRNA binding sites (RL-con) was used as a control. An FF construct without miRNA sites was used to normalize the transfection efficiency between the sets used for this assay (*n* = 3). Values obtained with anti-miR-122-transfected cells were taken as units. (F and G) Relative association of miR-125b and miR-142-3p with Ago2 after immunoprecipitation in LPS-treated control anti-miR-122- or anti-miR-146a-transfected cells (F). Reduced association of miR-125b target mRNA HIF1AN with Ago2 was also observed in anti-miR-146a-transfected cells (G). Macrophages were activated with LPS after transfection with the FHA-Ago2 expression construct and respective anti-miR inhibitor oligonucleotides. Western blot analyses of HA confirmed the amount of Ago2 pulled down postimmunoprecipitation that was used for normalization (*n* = 3). Values obtained with anti-miR-122-transfected cells were taken as units. (H) Association of miR-125b and miR-142-3p with endogenous Ago2 after immunoprecipitation with anti-Ago2 antibody from LPS-treated anti-miR-146a- or control anti-miR-22-transfected cells (*n* = 3). Values obtained with anti-miR-122-transfected cells were taken as units. (I and J) Relative levels of association of miR-125b and miR-142-3p with HA-Ago3 (I) and HA-Ago4 (J), respectively, after immunoprecipitation with anti-HA antibody from LPS-treated anti-miR-146a- or control miR-122-transfected cells. Western blot analyses of HA confirmed the amount of Ago variants pulled down postimmunoprecipitation that were used for normalization (*n* = 2). Values obtained with anti-miR-122-transfected cells were taken as units. (K) Reduced association of miR-21 with Ago2 after 24 h of LPS induction in anti-miR-146a-transfected cells. Macrophages have been activated with LPS after cotransfection with FHA-Ago2 expression plasmid and the respective anti-miR inhibitors (*n* = 3). Values obtained with anti-miR-122-transfected cells were taken as units. Immunoprecipitation was done with anti-HA antibody. (L) Levels of miR-21 target mRNAs after miR-146a inhibition determined by qRT-PCR in anti-miR-146a-transfected cells (*n* = 3). Values obtained with anti-miR-122-transfected cells were taken as units. (M) Derepression of miR-125b and miR-142-3p endogenous targets after 24 h of LPS induction in anti-miR-146a-transfected cells. qRT-PCR analyses of endogenous miR-125b targets (HIF1AN and MAP2K7) and miR-142-3p targets (ADCY9 and HMGB1) were done to estimate the relative mRNA content after anti-miR-146a transfection (*n* = 3). Values obtained with anti-miR-122-transfected cells were taken as units. (N) ChIP assay to study the relative enrichment of miR-125b and miR-142-3p endogenous target genes with RNA Pol II after 24 h of LPS induction in control anti-miR-122- or anti-miR-146a-transfected cells. Enrichment with IgG for the respective genes was used as a normalization control (*n* = 3). (O) miR-125b level does not affect cellular miR-146a-5p content. qRT-PCR data shows no effect of excess miR-125b on miR-146a level in murine macrophages transfected with miR-125b expression plasmid pmiR-125b against pCIneo control plasmid-transfected cells (*n* = 3). Values obtained with pCIneo plasmid-transfected cells were taken as units. (P and Q) Unaltered activities of miR-146a-5p on target mRNAs in pmiR-125b-transfected cells. Real-time PCR data show no significant change in the levels of endogenous targets of miR-146a-5p, TRAF6 and IRAK1, after miR-125b overexpression (*n* = 2). Decreased HIF1AN level confirmed increased activities of miR-125b after its overexpression (P). Dual-luciferase assay data exhibit no detectable change in miR-146a reporter RL-IRAK1 expression but increased repression of miR-125b reporter RL-MAD1 after miR-125b overexpression from pmiR-125 plasmid. Firefly luciferase (FF) constructs were used to normalize the transfection levels between the sets used in the assay (Q) (*n* = 3). Values obtained with control pCIneo plasmid-transfected cells were taken as units. 18s rRNA or GAPDH and U6 snRNA were used as endogenous targets in qRT-PCR of mRNA and miRNA quantification, respectively. Student’s *t* tests were used for all comparisons. *P* < 0.05 (*); *P* < 0.01 (**); *P* < 0.001 (***); *P* < 0.0001 (****).

To confirm the cooperative repressive activities of probable “coordinated groups of secondary miRNAs” in primary miRNA (miR-146a-5p)-compromised cells, we introduced a plasmid encoding RL reporter mRNA that contains MAD1L1-3′ UTR with binding sites for secondary miR-125b but without the primary miR-146a sites (30). The luciferase assay confirmed the miR-125b-dependent repression of RL-MAD1L1-3′ UTR having active miR-125b binding sites and the repression was rescued by anti-miR-125b treatment in both control and miR-146a-5p-expressing cells, where expression of miR-146-5p was induced from a doxycycline-responsive expression construct. These data suggest that the derepression of RL-MAD1L1-3′ UTR in the miR-146a-inhibited condition is due to the secondary miR-125b biogenesis defect rather than a direct miR-146a-mediated repression rescue (Fig. 4A, right panel). The luciferase assay also showed reduced repression of the miR-125b reporter target post-LPS induction in the anti-miR-146a-transfected murine macrophages (Fig. 4B). This observation has also been revalidated using reporters containing perfect binding sites for secondary miRNAs (Fig. 4C). Supporting the cooperativity among primary and secondary miRNAs for target repression, reporter constructs bearing binding sites for both miR-146a-5p and miR-125b showed better derepression than the reporter containing only the miR-146a site in anti-miR-146a-transfected murine macrophages (Fig. 4D and E). Ago2 is one of the key effector proteins of the RISC complex (31). An Ago2-based immunoprecipitation study showed a significant reduction in association with candidate miRNAs, namely, miR-125b and miR-142-3p, as well as secondary target mRNAs of miR-125b (HIF1AN), with Ago2 in LPS-induced macrophages transfected with miR-146a inhibitor oligonucleotides (Fig. 4F to H). Two other Ago variants, Ago3 and Ago4, expressed as hemagglutinin (HA)-tagged versions, also showed a similar trend of reduced association with secondary miRNAs in miR-146a-inhibited cells (Fig. 4I and J). As with miR-142-3p, miR-21 association with Ago2 also decreased, an increased expression of two miR-21 target mRNAs were noted in anti-miR-146a-treated cells (Fig. 4K and L). Consistent with the coordinated biogenesis (CB) effect, targets of miR-21, namely, PDCD4 and MAP2K3, were derepressed (Fig. 4L) (23, 32).

To explore the resonating effect of this CB phenomenon, we measured the effect on endogenous validated targets of those miRNAs generated as “secondary miRNAs” in CB. MAP2K7 and HIF1AN and ADCY9 and HMGB1 are all experimentally validated targets for miR-125b and miR-142-3p, respectively (33–36). There were no experimentally validated or predicted miR-146a-5p binding sites on the 3′ UTRs of those mRNAs, and thus their expression should not be affected directly by miR-146a-5p binding to the respective 3′ UTRs (27). Our observation revealed derepression of those “secondary” non-miR-146a-5p target genes upon miR-146a-5p inhibition in LPS-treated macrophages (Fig. 4M). In addition, we have also checked the influence of a possible transcriptional surge of secondary mRNA targets contributing to the observed changes in anti-miR-146a-treated cells. We performed a chromatin immunoprecipitation (ChIP) assay to study the differential enrichment of those secondary target genes with RNA polymerase II (Pol II). Our observation did not show any significant changes of RNA polymerase II enrichment of secondary target genes in anti-miR-146a-treated cells that negated the influence of transcriptional upregulation in miR-146a-inhibited LPS-induced macrophages (Fig. 4N). Overall, the observations depict a probable noncanonical function of miR-146a-5p, whereas a primary miRNA, miR-146a-5p, may cooperatively modulate the levels of secondary miRNAs and their target mRNAs, which do not harbor any identified miR-146a-5p binding site.

Does the miR-146a-5p-mediated biogenesis of secondary miRNAs and coordinated repression of secondary mRNAs have a feedback response on a “primary” miR-146a-5p level? We wanted to determine if there exists a reciprocal effect of a secondary miRNA, miR-125b, on the miR-146a-5p-mediated coordinated biogenesis function. To understand this, we introduced the miR-125b expression construct pmiR-125b into murine macrophages and investigated the alteration of miR-146a-5p expression and activity. A dual-luciferase assay confirmed the increased miR-125b biogenesis and activity after miR-125b expression (Fig. 4O to Q). We could not detect any significant change in miR-146a-5p level or repressive activity on the endogenous target or reporter mRNAs after miR-125b expression. These data nullify the probable miR-125b-mediated feedback responses on miR-146a-5p biogenesis and activity (Fig. 4O to Q). These findings led us to conclude that miR-146a-5p governing the CB operates unidirectionally for miR-125b in activated macrophages. The network is specific to miR-146a-5p, and differential expression of miR-125b-5p could not lead to consequent changes in activity and biogenesis of the miR-146a-5p. miR-146a-5p-mediated cooperativity is thus specific for a group of “secondary” miRNAs, and it probably acts unidirectionally.

### Dicer1-mediated miRNA processing imparts a crucial role for manifestation of TDCB.

**FIG 5:**
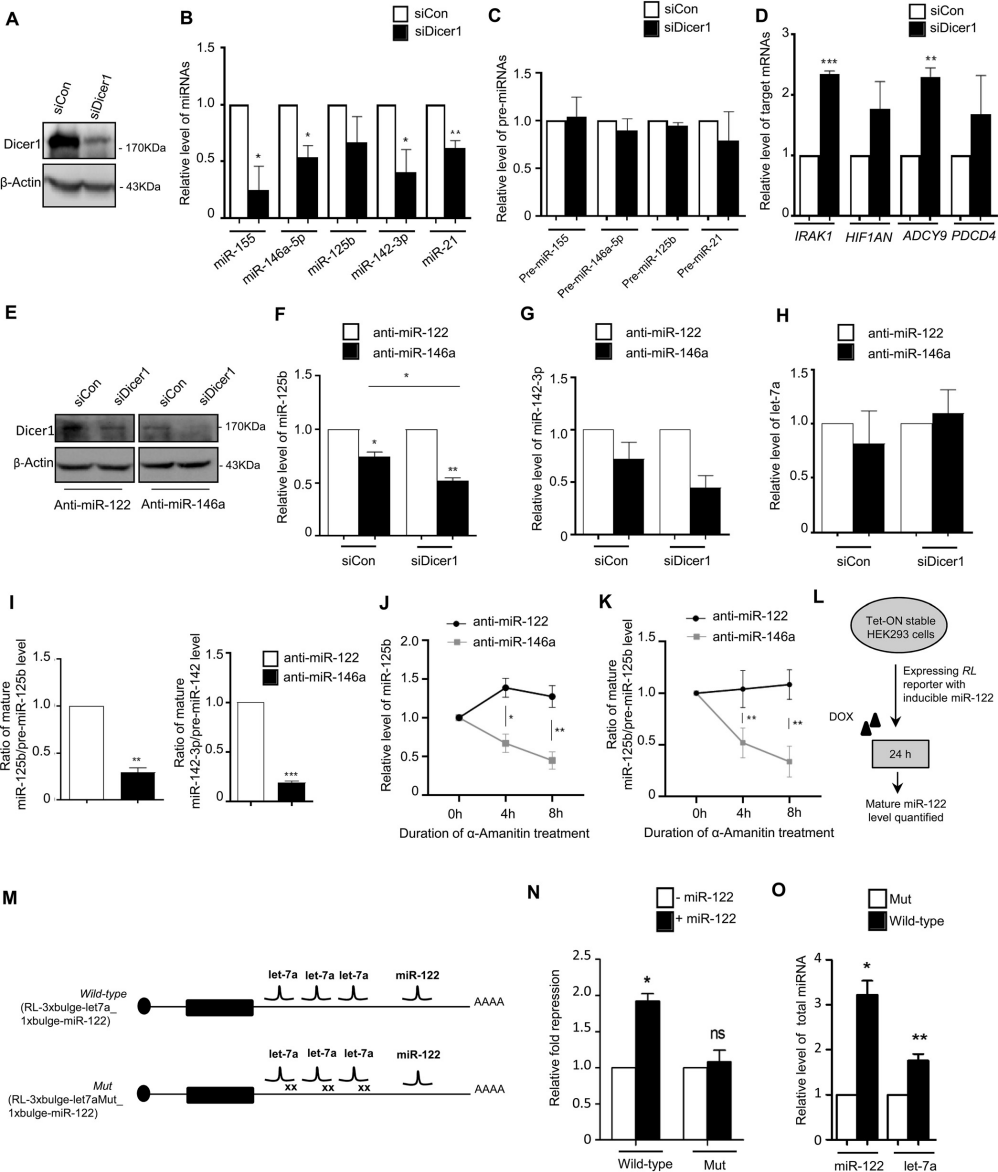
Dicer1-mediated processivity of secondary miRNAs drives TDCB. (A to D) Knockdown of Dicer1 inhibits LPS-induced upregulation of groups of LPS-responsive miRNAs. (A) Western blots show confirmation of Dicer1 knockdown in LPS-treated murine macrophages. Western blots show reduced level of Dicer1 after siDicer1-mediated knockdown in RAW264.7 cells. β-Actin served as an endogenous control. (B) qRT-PCR-based relative quantification shows reduced levels of miR-155, miR-146a, miR-125b, miR-142-3p, and miR-21 in Dicer1-knocked-down cells treated with LPS (4 h treatment for miR-155, 24 h treatment for other miRNA measurements) (*n* = 3). (C) qRT-PCR-based relative quantifications were used to measure the levels of respective precursor miRNAs after Dicer1 knockdown in LPS-treated cells at same time-points as in panel B (*n* = 2). (D) Levels of target mRNAs of the respective miRNAs were measured upon Dicer1 depletion by siDicer1 in LPS-activated macrophages. In all cases, *n* = 3. Values in control siRNA-transfected cells (siCon) were taken as units. (E to H) Dicer1 knockdown exhibits an additive impact on the TDCB phenomenon after inhibiting primary miRNA miR-146-5p. (E) Confirmation of Dicer1 knockdown in anti-miR-146a-treated LPS-induced cells. β-Actin serves as an endogenous control. (F and G) qRT-PCR-based relative quantification shows reduced cellular levels in the miR-146a-5p-inhibited condition for both miR-125b and miR-142-3p, which are further reduced in Dicer1-knocked-down cells. (H) qRT-PCR-based relative quantification confirmed an unchanged let-7a level under identical conditions (*n* = 3). Values in anti-miR-122-transfected cells were taken as units. (I) Ratio of cellular levels of miR-125b and miR-142-3p to their respective precursors quantified in anti-miR-146a- and control anti-miR-122-transfected LPS-treated cells (*n* = 3). (J and K) The relative level of miR-125b (J) and the ratio of miR-125b to its precursor (K) were quantified after 10 μg/mL α-amanitin treatment for the respective time points in anti-miR-146a- and anti-miR-122-transfected 24-h LPS-treated cells (*n* = 3). (L) Scheme of the experiments to test coordinated biogenesis of miRNAs in HEK293 cells. (M) Design of the reporter constructs used for testing coordinated biogenesis of miRNA by its targets in HEK293 cells. RL reporters with wild-type or mutant let-7a miRNA binding sites and miR-122 sites in their 3′ UTRs are shown. (N) miR-122 represses target mRNA bearing functional let-7a sites but not with mutated let-7a sites present in *cis* with a single miR-122 site. HEK293 cells were transfected with RL-3xbulge-let-7a_1xbulge-miR-122 or RL-3xbulgeMut_1xbulge-miR-122, with or without a doxycycline-inducible miR-122 expression construct, and fold repression was measured by a luciferase assay done with the above-mentioned reporters and a *Renilla* reporter without any miRNA binding sites (RL-con). Relative fold repression values are plotted by taking the values without miR-122 expression as units in both cases (*n* = 3). (O) Target-dependent increased biogenesis of let-7a and miR-122 after transfection with let-7a target site containing RL-3xbulge-let-7a_1xbulge-miR-122 construct but not with let-7a mutant sites bearing the construct in HEK cells expressing miR-122 in an inducible manner (*n* = 3). 18s rRNA or GAPDH and U6 snRNA were used as endogenous targets in qRT-PCR of mRNA and miRNA quantification, respectively. Student’s *t* tests were used for all comparisons. *P* < 0.05 (*); *P* < 0.01 (**); *P* < 0.001 (***); *P* < 0.0001 (****). The concentration of LPS and duration of LPS induction on macrophages were 10 ng/mL and 24 h, respectively, wherever not mentioned.

The RNase III endonuclease Dicer1 plays a prominent role in processing of mature miRNA from its precursor, and it has been reported to be an important player in target-driven biogenesis of the miRNAs in mammalian cells (3). To identify the role of Dicer1 in processing and generation of the LPS-induced “secondary” miRNAs, we knocked down Dicer1 and did not observe LPS-induced induction of secondary miRNAs in Dicer1-depleted cells (Fig. 5A and B). These data are consistent with the derepression of respective target mRNAs in Dicer1-depleted cells (Fig. 5D). However, the levels of precursor miRNAs do not change significantly for either primary or secondary miRNAs in Dicer1-depleted cells (Fig. 5C). Thus, Dicer1 is required for induced expression of the respective miRNAs that are expressed upon LPS treatment of macrophages. To understand the differential impact of Dicer1 on TDCB candidate miRNAs versus non-TDCB miRNAs, we knocked down Dicer1 in miR-146a-5p-impaired cells. We observed reduced levels of secondary miRNAs, namely, miR-125b and miR-142-3p, in anti-miR-146a (+siCon [ON-TARGETplus nontargeting pool])-transfected LPS-treated cells (Fig. 5E to G). Interestingly, the secondary miRNA level was further reduced when Dicer1 was knocked down along with miR-146a-5p inhibition in LPS-treated cells (Fig. 5F and G). Interestingly, our data did not show any significant changes for a non-TDCB candidate miRNA let-7a in either the anti-miR-146a-transfected control set or Dicer1 knockdown cells (Fig. 5H). These data depict an additive impact of Dicer1 on miR-146a-5p-mediated TDCB on secondary miRNAs that is absent for non-TDCB candidates of miRNA.

Interestingly, the ratio between the mature miRNA and pre-miRNA showed a decrease for the secondary miRNAs in anti-miR-146a-transfected cells (Fig. 5I). To ascertain the fact that this decreased level of secondary miRNA level is dependent on the decreased processivity of pre-miRs and not due to a decrease in transcription level, we monitored the levels of secondary miRNAs after blocking *de novo* transcription of primary miRNAs (pri-miRNAs) in anti-miR-transfected LPS-treated cells. Our observations showed a time-dependent decrease in miR-125b level in miR-146a-5p-inhibited cells after transcription inhibition, although the miR-125b level did not decrease in the control anti-miR-122 set (Fig. 5J). To strengthen the concept of CB and to confirm that the changes in miR-125b levels are not associated with a secondary miRNA degradation rate change, we measured the ratios of mature miRNA to pre-miRNA in anti-miR-146a- or control anti-miR-122-transfected LPS-treated cells after transcription inhibition. Interestingly, our observation showed a reduced level of pre-miRNA processivity evident in a relative decrease of the mature miR-125b to pre-miR-125b ratio in anti-miR-146a-transfected cells compared to anti-miR-122-transfected control sets after the blocking of transcription (Fig. 5K). These observations led us to conclude that the increase in secondary miRNA biogenesis is associated with increased pre-miRNA processivity at the penultimate stage of miRNA biogenesis in the presence of a “primary” miRNA.

### Target mRNA-driven cooperative biogenesis of miRNAs also occurs in non-macrophage cells.

To reconfirm the specificity and generality of our observation with miR-146a-5p and its downstream secondary miRNAs, for other primary miRNA-secondary miRNA pairs, we used let-7a and miR-122 as the primary and secondary miRNA pair in HEK293 cells to test our hypothesis in nonimmune mammalian cells. We designed an RL-encoding reporter mRNA bearing three bulged let-7a sites and a single miR-122 binding site i.e. RL-3xbulge-let-7a_1xbulge-miR-122, while an RL mRNA with mutated let-7a sites but with a single miR-122 site served as a control i.e. RL-3xbulge-let-7aMut_1xbulge-miR-122 (Fig. 5L and M). let-7a is endogenously expressed in HEK293 cells and miR-122 expression was induced by doxycycline from an miR-122 expression plasmid with a doxycycline-responsive promoter (Fig. 5L and M) (3). RL-3xbulge-let-7a_1xbulge-miR-122, but not RL-3xbulge-let-7aMut_1xbulge-miR-122, should be responsive to translation repression by let-7a, but repression by miR-122 should ideally operate for both constructs. Interestingly, when miR-122 was induced, repression by miR-122 was noted prominently for the RL-3xbulge-let-7a_1xbulge-miR-122 mRNA but not for the defective let-7a site-bearing construct (Fig. 5N). Quite predictably, and as shown earlier also, the let-7a sites on RL3xbulge-let-7a_1xbulge-miR-122 induced a target-dependent higher biogenesis of mature let-7a in HEK293 cells than the one having mutated let-7a sites (Fig. 5O) (3). Interestingly, miR-122 biogenesis was greatly enhanced only by RL-3xbulge-let-7a_1xbulge-miR-122 mRNA and not by the mutated let-7a binding site-containing construct. The overall observations restrengthen the TDCB phenomenon for another miRNA pair (let-7a and miR-122) to show that there is an increase in biogenesis and activity of secondary miRNA (in this case miR-122) with a smaller number of binding sites by a primary miRNA (in this case let-7a) with a greater number of target sites on the shared 3′ UTR of target mRNAs (in this case RL-3xbulge-let-7a_1xbulge-miR-122) in mammalian cells.

### Coordinate biogenesis relationship within miRNA-mRNA interactome in lipopolysaccharide-exposed macrophages.

**FIG 6:**
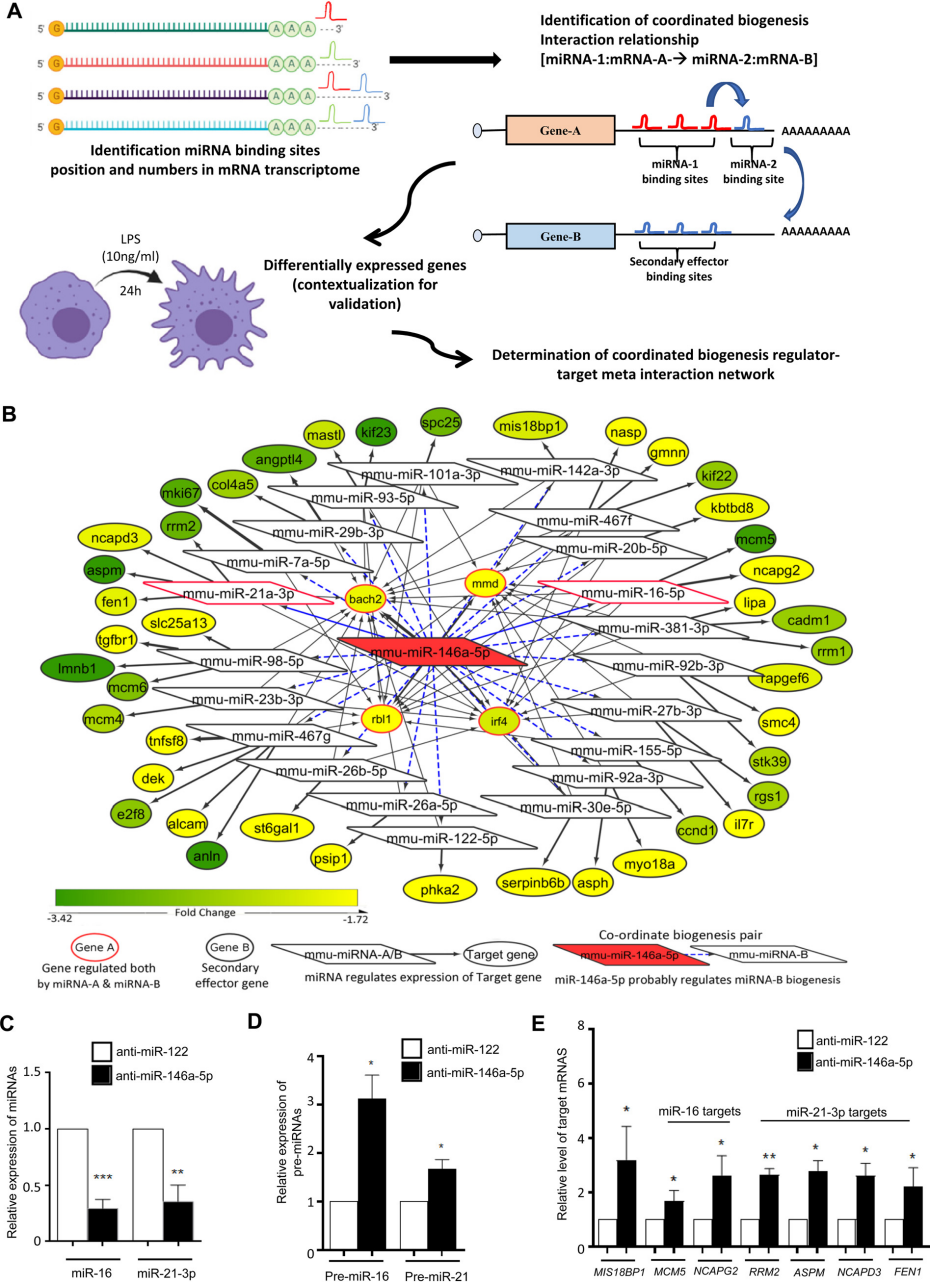
Network analyses and expression mapping reveal the group of miRNAs and their targets that are coordinately regulated by miR-146a-5p. (A) Schematic representation of the methodology utilized to predict CB regulator-target relationships for miR-146a-5p based on miRNA-mRNA network analysis. mRNA-A (gene A) bears two or more miR-146a-5p binding sites and fewer numbers of miRNA-2 (secondary miRNA) binding sites. Additionally, mRNA-B (gene B) harbors two or more miRNA-2 binding sites but no miR-146a-5p binding sites. (B) The set of predicted coordinate biogenesis relationships (miRNA-1:mRNA-A→miRNA-2:mRNA-B) considering mmu-miR-146a-5p as the regulatory miRNA is exemplified here. The fold change status of differentially expressed mRNAs in murine macrophages exposed to LPS (10 ng/mL) for 24 h (GEO accession no. GSE19490) was included in the analysis to identify downregulated gene A and gene B. (C to E) miR-146a-5p influences expression of miR-16 and miR-21-3p and their secondary effector mRNAs after LPS induction in anti-miR-146a-transfected murine macrophages. (C) qRT-PCR data confirm miR-16 and miR-21-3p level downregulation in anti-miR-146a-transfected LPS-treated RAW264.7 cells after 24 h of treatment. (D) Increased accumulations of their respective precursors are also evident in the same context. (E) Expressions of their target mRNAs were also found to be derepressed in the same cells. 18s rRNA or GAPDH and U6 snRNA levels were used as endogenous targets for real-time qRT-PCR-based mRNA and miRNA quantification, respectively. 18s rRNA or GAPDH and U6 snRNA were used as endogenous targets in qRT-PCR of mRNA and miRNA quantification, respectively. Student’s *t* tests were used for all comparisons. *P* < 0.05 (*); *P* < 0.01 (**); *P* < 0.001 (***); *P* < 0.0001 (****).

To explore the miR-146a-5p-dependent and target-mediated cooperativity in secondary miRNA biogenesis in a larger cellular context, we analyzed the differential expression of whole-cell transcriptomes after LPS-mediated stimulation of macrophages. We observed that miR-146a-5p may exhibit CB wherein it influences the generation of a cluster of miRNAs. Based on certain probable prerequisite conditions for the CB phenomenon, the possible CB regulator-target relationships of the CB regulator mmu-miR-146a-5p in lipopolysaccharide-exposed murine macrophages were predicted (Fig. 6A). Based on the assumptions considered for the CB phenomenon to occur, around 65,535 CB regulator-target (miRNA-1:mRNA-A coupled to miRNA-2:mRNA-B) relationships were sampled. These CB regulator-target relationships were obtained across 57 genes (“gene A”) and 187 numbers of miRNA-2 (“secondary miRNA”), considering mmu-miR-146a-5p (miRNA-1 or “primary” miRNA) as the CB regulator. Utilizing expression information to ascertain which genes are likely to be expressed during macrophage immune responses, we predicted the mmu-miR-146a-5p coordinate biogenesis regulatory network. Approximately 116 CB regulator-target relationships were determined in which both gene A and gene B were downregulated, given that the CB regulator and secondary effector miRNA in turn are likely to be upregulated. However, 91 CB regulator-target relationships were considered for further analysis, since there was no known or published direct regulatory relationship between gene A and gene B in those sets (Fig. 6B). These criteria resulted in a final set of 91 predicted CB regulator-target relationships for possible experimental validation in which both gene A and gene B were significantly downregulated (see Table S1 in the supplemental material). Thus, based on the assumptions of coordinate biogenesis with the help of our computational analysis, we identified 21 possible candidate secondary effector miRNAs (i.e., miRNA-2) whose expression may be regulated by miR-146a-5p because of CB. Further, it is possible that these 21 secondary effector miRNAs may subsequently regulate the expression of 42 mRNA species (from secondary effector genes) (Table S1). To confirm our hypothesis, we subsequently determined whether a change in the levels of the CB regulator is reflected in the levels of the secondary effector miRNA and its corresponding target mRNA in biochemical experiments. In order to validate whether the proposed computational methodology can indeed identify the biologically relevant CB regulatory network, candidate miRNAs were selected. Since *Bach2* (as gene A) possesses the highest number of miR-146a-5p binding-sites (4 in number), we have selected the associated set of CB regulator-target relationships for further analysis and validation. Our biochemical data showed significant downregulation of two potential miRNAs (namely, miR-16 and miR-21-3p) and increased accumulation of the precursor miRNAs after anti-miR-146a transfection in LPS-induced macrophages (Fig. 6C and D). Consequently, we could observe significant derepression in the predicted secondary effector genes as well, such as the *Mcm5* and *Ncapg2* (downstream targets of miR-16) and *Rrm2*, *Aspm*, *Ncadp3*, and *Fen1* (downstream targets of miR-21-3p) (Fig. 6E). Therefore, it is likely that a perturbation at the levels of the CB regulator (miR-146a-5p) affects the downstream targets in the coordinate biogenesis regulatory network, such as the secondary effector miRNA (miR-16, miR-21-3p, and miR-142-3p as previously shown) and their target genes. These data suggest that miR-146a-5p can indeed potentially exhibit CB for a group of miRNAs and regulate their activity in LPS-induced murine macrophages.

**FIG 7:**
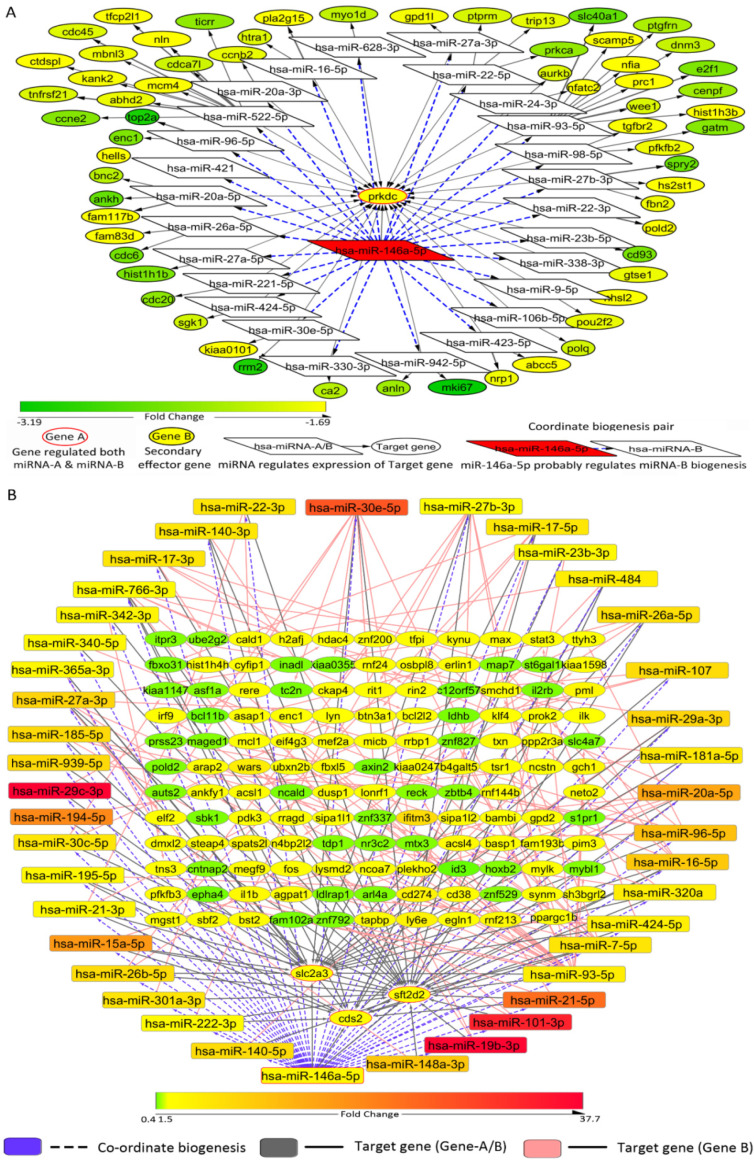
Possible CB regulator-target relationships considering hsa-miR-146a-5p as the regulator in the human system. (A) Considering hsa-miR-146a-5p as the regulatory miRNA (miRNA-1), the possible set of miRNA-1:mRNA-A→miRNA-2:mRNA-B coordinate biogenesis relationships have been exemplified here. The fold change status of differentially expressed mRNAs in macrophages exposed to LPS (10 ng/mL) for 24 h (GEO accession no. GSE85333) has been included for downregulated gene A and gene B. (B) CB regulator-target relationships in human monocyte-derived macrophages in response to Mycobacterium tuberculosis infection.

Moreover, we studied whether CB relationships between miRNAs can have species-specific differences. In order to investigate this possibility, we predicted the probable “CB relationships” that miR-146a-5p can have in human macrophages. Herein, initially by sampling the 33,214 probable CB regulator-target relationships that can occur in cells across 21 genes (gene A) and 616 miRNA-2s, a regulatory network was determined considering hsa-miR-146a-5p (miRNA-1) as the primary CB regulator. Since expression analysis may give us an idea regarding the context-dependent activation profiles of different precursor mRNAs, differential expression analysis was performed to determine which mRNAs are likely to get downregulated given that the CB regulator (miR-146a-5p) is upregulated upon LPS exposure and it influences the biogenesis of certain secondary effector miRNA/mRNA as well. Thus, a set of 62 CB regulator-target relationships were predicted in which both gene A and gene B were downregulated and wherein a direct regulatory relationship between gene A and gene B does not exist (Fig. 7A; Table S2). Subsequently, it would have been interesting to study whether a change in the concentration of mRNA-A is reflected in the concentration of the secondary effector mRNA (mRNA-B), as we could analyze mRNA expression profiles in LPS-stimulated human monocytes. Herein, we observed that miR-16 can act as a secondary effector miRNA in the miR-146a-5p regulatory network responding to LPS exposure in human macrophages as well (Fig. 7A; Table S2). Further, some miRNAs (miR-16, miR-27b, miR-26a, miR-30e, miR-93, and miR-98) are likely to be regulated by miR-146a-5p in a similar manner in human monocytes and murine monocytes following LPS stimulation. However, there exist some species-specific differences based on the organization of the miRNA binding sites in tandem in genes. This species-specific variation in the organization of miRNA binding sites is a possible explanation behind the species-specific variances in the networks (Fig. 6B and Fig. 7A). Interestingly, the miR-146a-5p CB regulatory network in macrophages may exhibit condition-specific differences in regulatory network components. For instance, in macrophages responding to LPS stimuli or Mycobacterium tuberculosis infection, a large set of different miRNAs are likely to be involved, while a fraction of miRNAs (miR-16, miR-20a, miR-27a, miR-27b, miR-26a, miR-22, miR-424, miR-30e, miR-93, and miR-96) were same among these networks (Fig. 7A and B; Table S3). Further, most of these predicted secondary effector miRNAs, particularly miR-16, miR-23b, miR-26a, miR-27b, miR-30e, miR-93, and miR-98, are known to modulate immune responses or inflammatory responses (16, 37). Therefore, by studying the CB regulatory network of miR-146a-5p under different contexts, we determined that these miRNAs along with their secondary effector miRNAs (miR-16, miR-21, miR-29b, or miR-93) can potentially concordantly regulate and fine-tune macrophage inflammatory responses.

### Activation of macrophages is not a prerequisite for miR-146a-5p-mediated coordinated biogenesis of secondary miRNAs.

**FIG 8:**
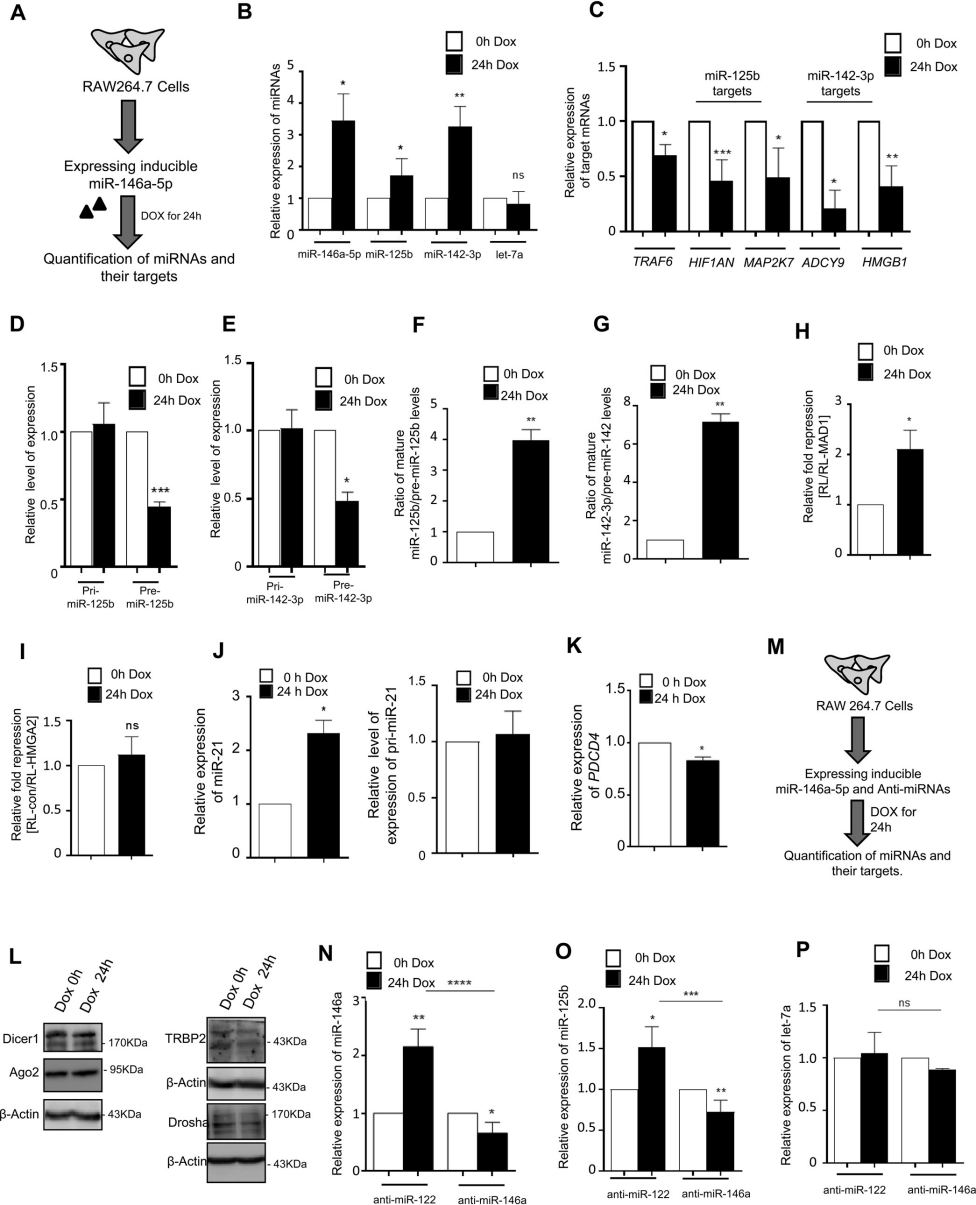
miR-146a-5p could promote coordinated biogenesis of secondary miRNAs in non-LPS-activated murine macrophages. (A) Scheme of experiment to study miR-146a-5p-mediated coordinated biogenesis of miRNAs in Tet-On RAW264.7 cells. (B) Levels of miR-125b and miR-142-3p in cells expressing miR-146a-5p in an inducible manner in the presence of doxycycline. Relative levels of miRNAs were measured after treatment with 400 ng/mL of doxycycline for 24 h. qRT-PCR data confirm induced expression of miR-146a-5p and coordinated upregulation of two candidate miRNAs, miR-125b and miR-142-3p. Let-7a, a non-CB pair miRNA of miR-146-5p, did not show any change in Tet-On RAW264.7 cells after miR-146a induction (*n* = 3). (C) Expression of endogenous targets of miR-125b and miR-142-3p in RAW264.7 cells expressing miR-146a-5p in the presence of doxycycline. The qRT-PCR-based quantification also confirmed the increased miR-146a-mediated repression of its target TRAF6 after inducible expression of miR-146a-5p (*n* = 3). (D and E) Cellular levels of primary (pri-) and precursors (pre-) of miR-125b or miR-142-3p in RAW264.7 cells expressing miR-146a-5p in the presence and absence of doxycycline (*n* = 3). Values in non-doxycycline-treated cells are taken as units. (F and G) Ratios of cellular levels of miR-125b and miR-142-3p to their respective precursors were quantified in RAW264.7 cells expressing miR-146a-5p in the presence of doxycycline (*n* = 3). Values in non-doxycycline-treated cells are taken as units. (H and I) Repressive activity of miR-125b after induction of miR-146a-5p in RAW264.7 macrophage. A dual-luciferase assay was done with RL-MAD1 reporter mRNA having miR-125b binding sites in its 3′ UTR. RL-MAD1 but not RL-HMGA2, which harbors the 3′ UTR of HMGA2 with seven let-7a sites on its 3′ UTR, showed repression in cells expressing miR-146a-5p after 24 h of doxycycline induction. RL reporters without miRNA sites were used as a control. Firefly luciferase acts as a normalization control (*n* = 3). (J) The level of miR-21 is increased upon inducible expression of miR-146a-5p in macrophages (left panel). The relative level of pri-miR-21 was unchanged under the same condition (right panel). (K) PDCD4, an endogenous target of miR-21, was found to be repressed in cells expressing miR-146-5p (*n* = 3). (L) miRNP-associated proteins do not show alteration in expression upon inducible miR-146a-5p expression in RAW264.7 macrophages. miRNP-associated proteins and processing enzymes Dicer1, Ago2, TRBP2, and Drosha did not show any significant change in their expression in miR-146a-5p-induced cells. (M) Experimental scheme that is followed for experiments described for panels N to P, where anti-miRs and the doxycycline-inducible miR-146a-5p construct were cotransfected in Tet-On RAW264.7 cells. (N to P) Effect of miR-146a-5p inhibition in Tet-On RAW264.7 cells expressing miR-146a-5p in an inducible manner. Reduced miR-146a-5p (N), miR-125b (O), and unchanged let-7a (P) levels in miR-146a-5p-expressing cells in the presence of miR-146a inhibitor but not in the presence of anti-miR-122 in Tet-On RAW264.7 cells after 24 h of doxycycline treatment are shown. 18s rRNA or GAPDH and U6 snRNA levels have been used as an endogenous target for real-time qRT-PCR of mRNA and miRNA quantification, respectively. Student’s *t* tests were used for all comparisons. *P* < 0.05 (*); *P* < 0.01 (**); *P* < 0.001 (***); *P*< 0.0001 (****).

Further, we wanted to explore the exclusive contribution of miR-146a-5p to the biogenesis of secondary miRNAs irrespective of endotoxin-induced activation status of RAW264.7 macrophages. miR-146a-5p may have a prominent role, but thousands of different LPS-responsive molecules may also impart a crucial additive effect to miR-146a-5p-mediated TDCB. To address the notion and to understand the exclusive role of miR-146a-5p in TDCB, we adopted an miR-146a-5p overexpression system in naive macrophages without LPS treatment. We adopted a doxycycline-responsive (Tet-On) miR-146a-5p expression system to validate our observations independently of macrophage activation by LPS (Fig. 8A). Inducible expression of miR-146a resulted in significant upregulation of miR-125b and miR-142-3p after 24 h of doxycycline induction (Fig. 8B), but as with our previous observations, we could not see a change in the level of the non-CB pair miRNA let-7a upon miR-146a-5p induction (Fig. 8B). Corroborating this, repression of endogenous targets of potential secondary miRNAs, miR-125b and miR-142-3p, could also be observed (Fig. 8C). Consistent with TDCB, the cellular level of secondary pre-miRNAs was found to be decreased while an unchanged level of pri-miRNAs correlated with increased mature miRNA processing. These data also neglect a possible transcriptional surge for secondary pri-miRNAs after miR-146a overexpression (Fig. 8D and E). In addition, we also measured the ratio of mature secondary miRNAs to secondary pre-miRNAs. The data suggests increased pre-miRNA processivity for secondary miRNAs (of miR-125b and miR-142-3p) in miR-146a-overexpressing cells and revalidates our hypothesis for CB (Fig. 8F and G). Dual-luciferase reporter assay data further strengthened our observations that showed an increased activity for miR-125b but not for non-CB miRNA let-7a (Fig. 8H and I) in miR-146a-5p-overexpressing cells. For miR-21, the other candidate secondary miRNA, we could see upregulation of the miR-21 level and repression of its target PDCD4 after miR-146a-5p induction in macrophages (Fig. 8J and K). We wanted to check the levels of different microribonucleoprotein (miRNP)-associated proteins post-miR-146a induction to rule out their possible contribution to the increased miRNP production observed for secondary miRNAs in miR-146a-5p-expressing cells. However, we did not observe any significant change in expression of those factors in miR-146a-5p-expressing cells. This result nullifies the possibilities of miR-146a-5p-mediated upregulation of an miRNP-specific factor in modulation of the overall miRNA level inside the cells (Fig. 8L). To further rule out any other mode of cross-regulation other than miR-146a-5p-mediated cooperativity in biogenesis of miR-125b, we used the doxycycline-responsive (Tet-On) miR-146a-expressing system in the presence of anti-miR-146a-5p in macrophages to determine the role of miR-146a-5p in TDCB of miR-125b (Fig. 8M). We could see a synchronized and coordinated expression of miR-125b along with miR-146a-5p in macrophages (Fig. 8N and O). We could also observe that the cellular level of miR-125b relates to miR-146a abundance in macrophages after the addition of doxycycline, and we could see no upsurge of miR-146a-5p or miR-125b level in the miR-146a-inhibited environment (Fig. 8N and O). To understand the specificity of miR-125b expression by miR-146a-5p, we measured let-7a levels, and we could not see any changes in expression level of non-CB pair miRNA let-7a under the same conditions (Fig. 8P). Together, these data strengthen the fact that miR-146a-5p orchestrates a key step to cooperatively modulate the biogenesis and activities of groups of secondary miRNAs in mammalian macrophages.

### Small-RNA sequence analysis reveals the coordinated biogenesis network of miR-146a-5p.

The computational methodology proposed herein based on binding site analysis provided indications regarding which miRNAs (secondary effector) are likely to participate in the miR-146a-5p-driven coordinate biogenesis network formation. Considering all possible combinations, it is possible that miR-146a-5p can influence the expression of multiple secondary effector miRNAs (187) when it acts as an miRNA biogenesis regulator, considering the existence of CB phenomenon. In order to understand whether this phenomenon can have large-scale implications for the global miRNA-mRNA interaction networks within cells, we performed a sequencing analysis to determine the likely secondary effector miRNAs that exhibited a change in their expression profiles when the CB regulator miR-146a-5p was expressed in an inducible manner. Herein, we identified 109 miRNAs that were differentially expressed because of miR-146a-5p overexpression in macrophage cell lines. Among the miRNAs that were differentially expressed in the mature state, 44 miRNAs showed upregulation while 65 miRNAs exhibited downregulation (Table S4).

Considering the miRNA expression profiling data, we suggested that miR-146a-5p is likely to influence the biogenesis of mmu-miR-16-5p, mmu-miR-23b-3p, mmu-miR-150-5p, mmu-miR-15b-5p, mmu-miR-25-3p, mmu-miR-322-5p, mmu-miR-34a-5p, and mmu-miR-500-3p. Further, we could validate the presence of secondary effector miRNAs (mmu-miR-23b-3p and mmu-miR-16-5p) in the miR-146a-5p regulatory network, since these miRNAs showed significant upregulation in their mature forms. However, some predicted secondary effector miRNAs (mmu-miR-7a-5p) were found to be downregulated as well. Additionally, considering our previous analysis of the macrophage response to LPS exposure, 11 CB regulator-target relationships (Table 1) were determined wherein miR-16-5p or miR-23b-3p (miRNA-2) biogenesis is likely to be influenced (upregulated) by coordinated biogenesis and both primary and secondary effector mRNAs (gene A, gene B) are downregulated.

**TABLE 1:** CB regulator-target relationships likely to occur in miR-146a-5p coordinate biogenesis regulatory network in murine macrophages responding to LPS exposure

Gene with tandem miRNA pairs (gene A)	CB pair regulator (miRNA-1)	Target gene of secondary effector miRNA (gene B)	Secondary effector miRNA (miRNA-2)	logFC^*a*^ (miRNA-2)	*P* value^*b*^ (miRNA-2)	Counts of miRNA CB pairs (prevalence)
*bach2*	mmu-miR-146a-5p	*mcm5*	mmu-miR-16-5p	0.611762	0.000437	39
*mmd*	mmu-miR-146a-5p	*mcm5*	mmu-miR-16-5p	0.611762	0.000437	39
*rbl1*	mmu-miR-146a-5p	*mcm5*	mmu-miR-16-5p	0.611762	0.000437	39
*irf4*	mmu-miR-146a-5p	*mcm5*	mmu-miR-16-5p	0.611762	0.000437	39
*bach2*	mmu-miR-146a-5p	*ncapg2*	mmu-miR-16-5p	0.611762	0.000437	39
*mmd*	mmu-miR-146a-5p	*ncapg2*	mmu-miR-16-5p	0.611762	0.000437	39
*rbl1*	mmu-miR-146a-5p	*ncapg2*	mmu-miR-16-5p	0.611762	0.000437	39
*irf4*	mmu-miR-146a-5p	*ncapg2*	mmu-miR-16-5p	0.611762	0.000437	39
*irf4*	mmu-miR-146a-5p	*mcm4*	mmu-miR-23b-3p	0.295528	0.02836	21
*bach2*	mmu-miR-146a-5p	*mcm4*	mmu-miR-23b-3p	0.295528	0.02836	21
*rbl1*	mmu-miR-146a-5p	*mcm4*	mmu-miR-23b-3p	0.295528	0.02836	21

a logFC, fold change in secondary effector miRNA in miR-146a-overexpressing RAW264.7 cells compared to the control.

b *P* value, significance of fold change expression in secondary effector miRNA in miR-146a-overexpressing RAW264.7 cells compared to the control.

Subsequently, we wanted to determine the probable physiological scenarios that are likely to be driven by the miR-146a-5p cooperative biogenesis network. In this respect, we identified validated secondary effectors like miR-16 and miR-23b that are known to modulate immune responses or inflammatory responses (16, 37). The target genes of the probable secondary effector miRNAs (mmu-miR-16-5p, mmu-miR-23b-3p, mmu-miR-150-5p, mmu-miR-15b-5p, mmu-miR-25-3p, mmu-miR-322-5p, mmu-miR-34a-5p, and mmu-miR-500-3p) identified both in the sequencing analysis and the computational analysis along with predicted target genes of mmu-miR-146a-5p were considered for pathway enrichment analysis. KEGG pathway enrichment analysis identified the Salmonella infection “mmu05132” pathway as enriched, and enriched terms herein were mainly found to occur in different signaling pathways like the NF-κB, MAPK, or apoptosis pathway (Table 2 and data not shown).

**TABLE 2:** Kegg pathway enrichment analysis of target genes of miR-146a and secondary effector mRNA in miR-146a coordinate biogenesis network

ID	Description	Gene ratio	Bg ratio^*a*^	*P* value	Gene ID	Count
mmu05132	Salmonella infection	15/170	253/8900	9.57E−05	140580/18033/18481/192656/19766/20336/20430/234663/235406/235661/23797/50884/66713/66724/67166	15

a Bg ratio, background ratio that refers to numbers on genes annotated to a pathway within a background set.

### Coordinated biogenesis of miRNA affects cellular signaling pathways to protect cells from LPS-induced inflammation and apoptosis.

**FIG 9:**
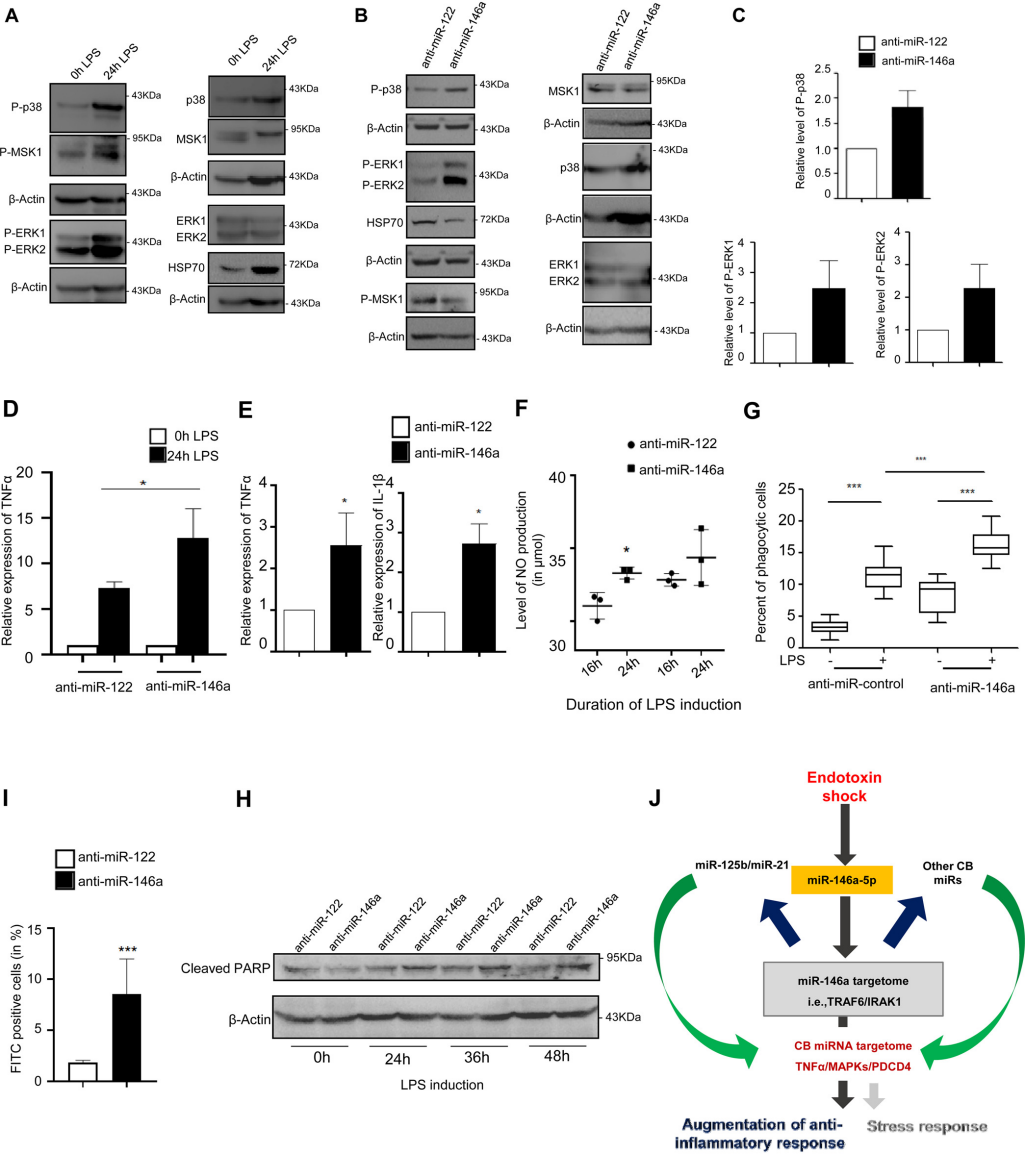
Targeting of primary miRNA miR-146a-5p affects the cellular response in LPS-activated RAW264.7 cells. (A) Induction with LPS causes activation of MAPK signaling molecules. (B) Inhibition of miR-146a-5p activity leads to overactivation of MAPK signaling molecules. Representative Western blot data confirm the increased phosphorylation status of P-p38 and P-ERK1/2 molecules upon LPS treatment in anti-miR-146a-treated but not in control anti-miR-122-treated RAW264.7 macrophages. Cellular levels of HSP70 and P-MSK1 were also measured. p38, ERK1/2, and MSK1 were also detected in anti-miR-146a and control anti-miR-122-transfected cells. β-Actin served as an internal control. (C) Comparative densitometric analysis data obtained from Western blots were plotted to show the changed phosphorylation status of MAPK signaling molecules P-p38 and P-ERK1/2 in anti-miR146a-treated LPS-induced cells compared to control anti-miR-122-transfected cell sets. (D and E) Effect of anti-miR-146a treatment on LPS-induced expression of TNF-α levels in RAW264.7 cells. (D) Relative levels of proteins were measured by ELISA and plotted for both anti-miR-146a- and anti-miR-122-transfected cells. (E) Changes in levels of proinflammatory cytokine TNF-α (left panel) and IL-1β (right panel) mRNAs after 24 h of LPS induction in anti-miR-146a-transfected RAW264.7 murine macrophages. Levels in control anti-miR-122-transfected cells were considered as units. (F) Increased generation of nitric oxide in cells with reduced miR-146a activity upon LPS treatment. Griess assay-based quantification revealed increased NO production in anti-miR-146a-transfected cells compared to control anti-miR-122-transfected cells after 16 h and 24 h of LPS treatment (*n* = 3). (G and H) Inhibition of miR-146a activity leads to increased levels of phagocytosis and apoptosis in LPS-treated murine macrophages. (G) The percentage of phagocytosis was measured using fluorescence-labeled latex bead entry, and data were plotted. (H) miR-146a-5p plays a crucial role in combating LPS induced apoptosis in RAW264.7 cells. Western blot data confirm an increased cellular level of cleaved PARP in anti-miR-146a-transfected macrophages. (I) The percentages of apoptotic cells were calculated in a TUNEL assay done for anti-miR-122- and anti-miR-146a-transfected LPS-induced murine macrophages (n = 3). (J) Proposed coordinated biogenesis-regulated inflammatory network (CBIN) that operates inside activated macrophages to balance the immune response to protect cells from overactivation and cellular death. Blue and green arrows represent the TDCB phenomenon in murine macrophages. The 18s rRNA or GAPDH level has been used as an endogenous target for real-time qRT-PCR of mRNA quantification. Concentrations of LPS for induction on macrophages were 10 ng/mL for the indicated time points. Student’s *t* tests were used for all comparisons. *P* < 0.05 (*); *P* < 0.01 (**); *P* < 0.001 (***).

LPS induction leads to activation of multiple signaling pathways like the MAPK or apoptosis signaling pathway (Fig. 9A). Although many of the molecular mediators are known, little is known about the miRNA-mediated cross talk controlling these pathways. Previous reports suggest that upregulation of miR-146a/b acts as a compensatory response to curb inflammatory responses by targeting IRAK1 (of IL-1 receptor signaling) or TRAF6 or decreasing TNF-α production to form a negative feedback loop (16, 26, 38) in macrophages. The IRAK1/TRAF6 cascade signaling was known to activate p38 in a reactive oxygen species (ROS)-dependent manner as well as phosphorylation of extracellular signal-related kinases (39, 40). This intrigued us to investigate the differential expression of various signaling molecules of MAPKs in the absence of miR-146a-5p. We could observe an increased phosphorylation status of p38 and ERK1 molecules in the absence of miR-146a-5p activity on LPS-induced macrophages (Fig. 9B and C). In contrast, we could also observe a decreased level of HSP70 protein after anti-miR-146a transfection. Activation of HSP70 through LPS was known to be involved in the inhibition of NF- kβ activation, thus downregulating proinflammatory cytokine production (41, 42). We also checked the activated level of MSK1, a negative regulator of Toll-like receptor signaling, which acts downstream of p38 and ERK1/2 mitogen-activated protein kinases (43). Decreased levels of phosphorylated MSK1 were observed under miR-146a-5p-inhibited conditions (Fig. 9B). A significant increase in TNF-α and IL-1β levels has also been documented in the absence of miR-146a-5p activity, revalidating the reciprocal relationship it has with proinflammatory cytokine production (Fig. 9D and E). LPS induction also leads to production of inducible nitric oxide synthase (iNOS) expression, which ultimately results in increased NO production (44). Our data reveal increased generation of nitric oxide (NO) after anti-miR-146a transfection at different time intervals after LPS treatment (Fig. 9F). It is also evident that LPS-induced TNF-α and nitric oxide upregulation aid in initiation of apoptosis in macrophages (45). We were interested to check the level of the important apoptotic marker protein PARP post-anti-miR-146a transfection. PARP is a downstream molecule in the apoptosis pathway that is cleaved to generate an 89-kDa fragment in apoptotic cells (46). We could observe increased cellular cleaved PARP post-anti-miR-146a transfection at different LPS treatment time points (24 h, 36 h, 48 h) (Fig. 9H). Corroborating this, terminal deoxynucleotidyltransferase-mediated dUTP-biotin nick end labeling (TUNEL) assay data showed increased apoptosis in anti-miR-146a-transfected LPS-induced macrophages after 48 h (Fig. 9I). In addition, we quantified the level of phagocytosis of target macrophages using fluorescent latex beads. We could see increased levels of phagocytosis in miR-146a-inhibited LPS-activated macrophages (Fig. 9G). Thus, miR-146a-5p acts as a crucial regulator with respect to the neutralization of inflammatory responses that act through multiple pathways, be it miRNA-mediated CB or the secondary target molecule-mediated modulation of associated factors, which imparts a huge role in combating pathogenic responses. This led us to propose a CB-regulated inflammatory network model that probably exists inside the activated macrophages (Fig. 9J).

### COORD-BIO web server predicts CB regulator-target relationships in humans and mice.

**FIG 10:**
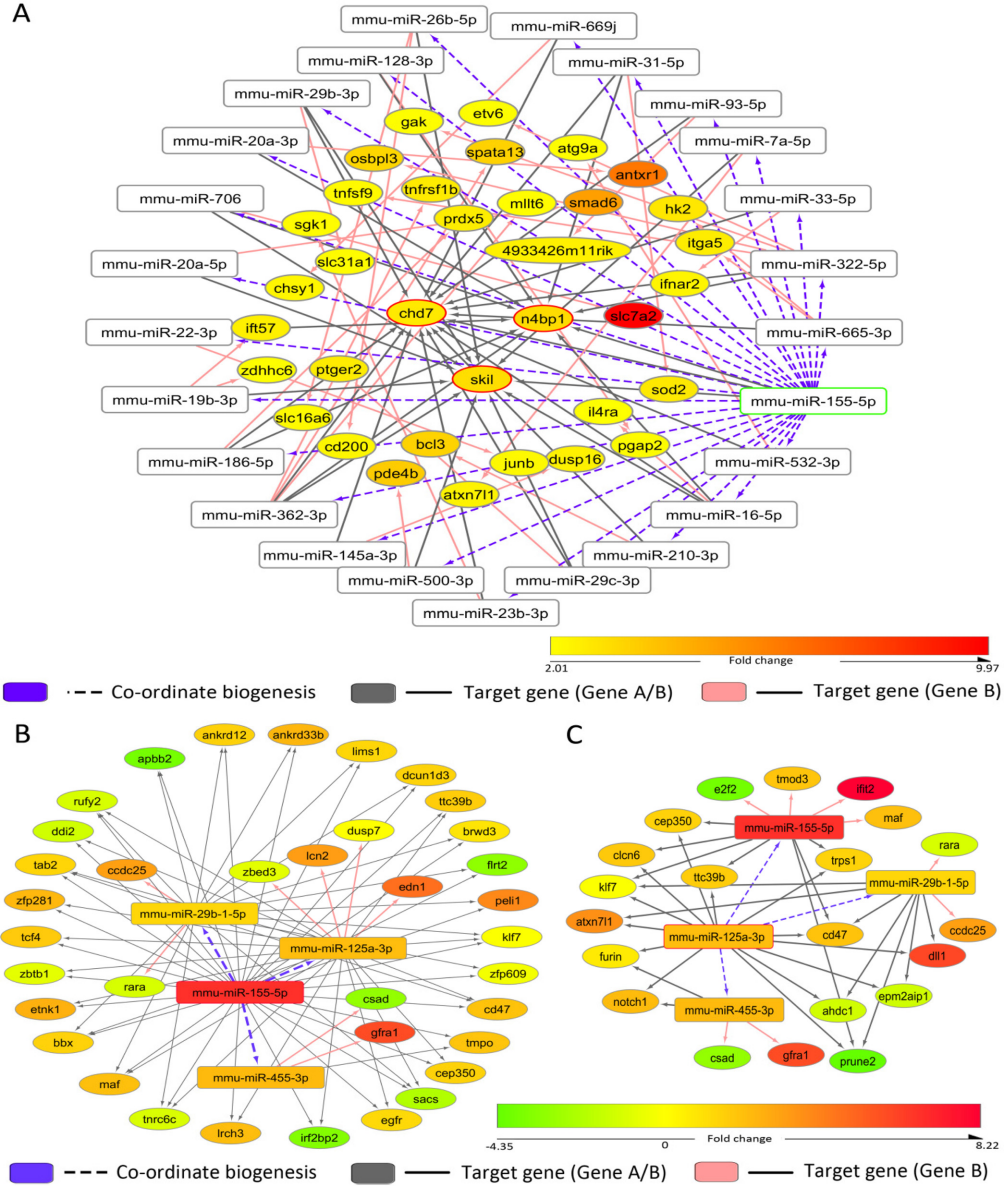
Possible CB regulator-target relationships considering mmu-miR-155 as the regulator are shown. A set of predicted coordinate biogenesis relationships (miRNA-1:mRNA-A→miRNA-2:mRNA-B) considering mmu-miR-155-5p as the regulatory primary miRNA is exemplified here. (A) Possible coordinate biogenesis network of miR-155-5p in murine macrophage under LPS exposure using miR-155 knock-out macrophage cells. (B) Possible coordinate biogenesis network of miR-155-5p in macrophage polarization. (C) Possible coordinate biogenesis network of miR-125a/b in hematopoietic cells.

Our previous analyses suggested that miR-146a-5p can regulate the biogenesis of specific miRNAs (particularly miR-16, miR-21, miR-142-3p, and miR-125b) and the associated secondary effector mRNAs in response to LPS-based stimulation of macrophage cell surface receptors. In this manner, a CB regulator may alter the expression profile of specific miRNAs, with mRNAs in turn altering or readjusting cellular miRNA-mRNA interaction networks as a result of the coordinate biogenesis phenomenon. Therefore, we next posed the question as to whether miRNAs other than miR-146a-5p can exhibit this coordinate biogenesis phenomenon. In this respect, we have predicted the CB regulator-target relationships for miR-155-5p and have determined whether there are corresponding changes in secondary effector miRNAs that are not directly regulated by miR-155-5p. Comparative transcriptional profiles of stimulated M1 (LPS plus gamma interferon [IFN-γ]) miR-155 knockout (KO) macrophages and unstimulated M0 KO macrophages (GEO accession no. GSE77425) were studied (47). Here, we determined that the target genes of miR-155 and the target genes of secondary effector miRNAs (miR-16-5p, miR-29b, or miR-322) are also differentially expressed. Thus, based on our predicted CB regulator-target relationship for miR-155, it is possible that miR-155 could potentially regulate the expression of secondary effector miRNAs thereby modulating the response of macrophages upon LPS exposure (Fig. 10A; Table S5). Similarly, miRNA-mRNA expression profiles in M1 and M2 polarized macrophages (48) also suggested that CB regulator (miR-155) and secondary effector miRNAs (miR-29b, miR-125a, miR-455) are simultaneously but differentially expressed there (Fig. 10B; Table S5). In some CB regulatory networks, we observed that there is a possibility of a reciprocal regulation or interaction (among miR-125a and miR-155 in macrophage polarization) as well (Fig. 10C; Table S5). Therefore, different miRNAs can concordantly exhibit the CB phenomenon to regulate the macrophage immune response in a particular scenario. However, CB is likely to occur in different cell types as well. In this respect, we again predicted the CB regulator-target relationships for miR-125a-3p by considering a condition in which the miRNA has been overexpressed to determine whether there are corresponding changes in secondary effector miRNAs that are not directly regulated by these miRNAs. CB regulator-target relationships for miR-125a-3p in hematopoietic cells during cell maturation were predicted utilizing a data set (GEO accession no. GSE33691) (49) wherein miR cluster 99b/let-7e/125a had been overexpressed. It was observed that miR-125a-3p likely influences the biogenesis of miR-125b-5p, miR-338-3p, miR-706, and miR-762 since gene A and associated secondary effector genes (19 mRNAs as gene B) are differentially expressed when the miR cluster 99b/let-7e/125a is overexpressed (Table S5). These secondary effector genes are not directly regulated by the miRNAs that are overexpressed but are indirectly regulated via intermediate miRNAs whose expressions are likely varying because of the CB phenomenon. Although extensive biochemical and multifaceted approaches could confirm the existence of CB relationships for a specific scenario, it is likely that the coordinate biogenesis phenomenon may exist in different physiological pathways as well to act as an additional important modulator for the fine-tuning of various cellular responses.

**FIG 11:**
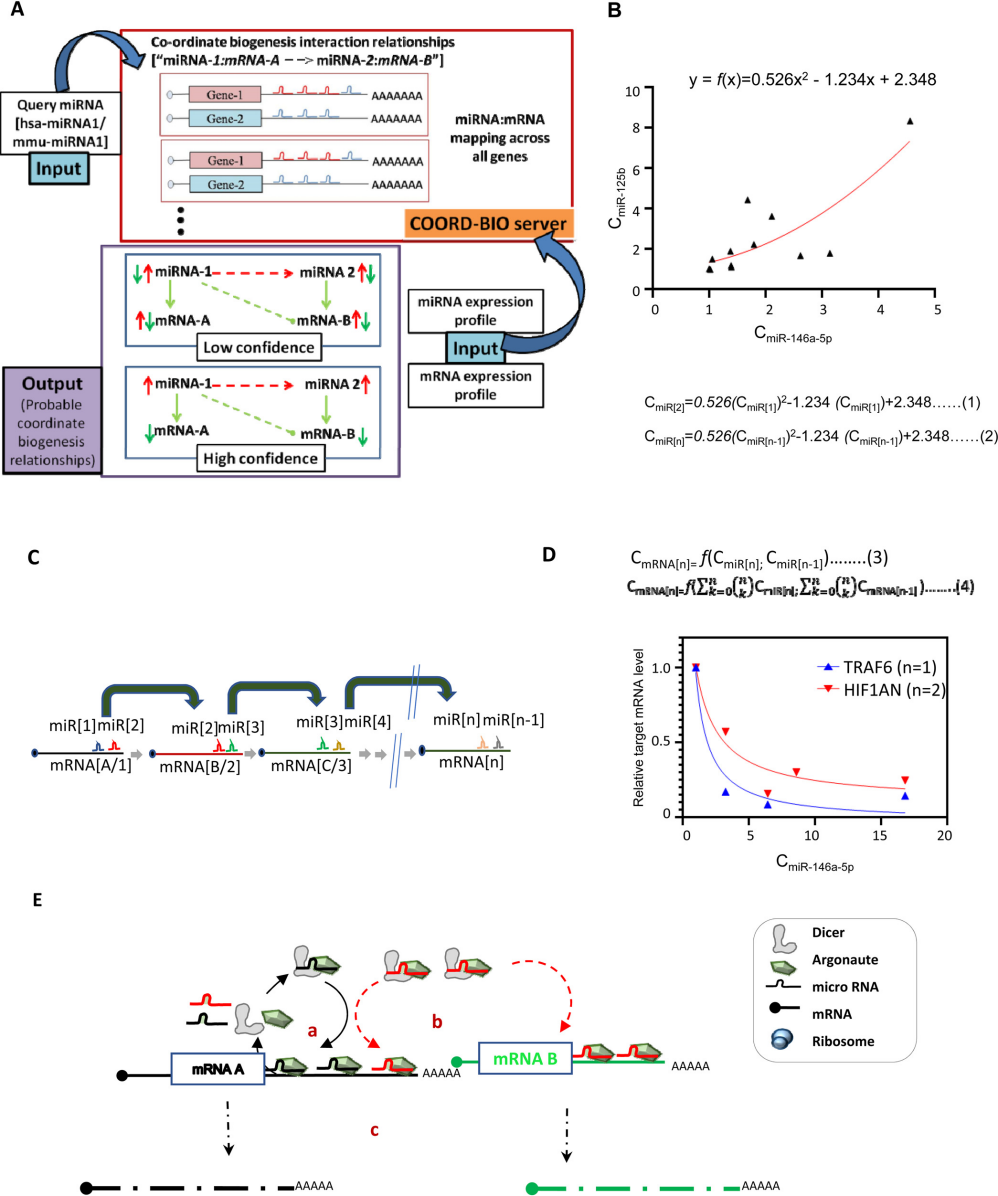
Progressive dampening mode of action of primary miRNAs on secondary targets. (A) Schematic representation of the COORD-BIO web server that provides information regarding coordinate biogenesis relationships in Homo sapiens and Mus musculus. COORD-BIO is available at http://www.hpppi.iicb.res.in/coordB2/index.html. (B) Change in expression of miR-125b in response to changing concentration of miR-146a-5p in RAW264.7 cells expressing the miR-146-5p in an inducible manner. The curve fitting of the data points is represented by a polynomial regression equation (equation 1) considering miRNA-125b as a secondary miRNA. The expression of an miRNA in a specific regulatory layer (*n*th layer) should be identified by the proposed equation (equation 2). (C) Schematic representation of target-dependent coordinated regulation of miRNA genesis showing how the concentration of miRNAs is probably being regulated through multiple layers. (D) Probable relation of a specific mRNA concentration with the levels of its primary and secondary miRNAs (equations 3 and 4). Plotting of data for two targets, TRAF6 (primary target of miR-146a-5p) and HIF1AN (primary target of secondary miRNA, i.e., miR-125b, and coordinated biogenesis-mediated secondary target of miR-146a-5p), against primary miRNA miR-146a-5p concentration. This data suggests a dampening effect of an miRNA on their non-“primary”targets, and the effect is reduced in an exponential way with the distance between the subsequent layers which the miRNA and the target mRNA belong to, respectively. (E) Graphical model of cooperative biogenesis of miRNAs in mammalian cells. In step a, target mRNA (mRNA A)-dependent cognate miRNA (miRNA 1 in black) biogenesis occurs, and in step b, the newly generated miRNP1 (Ago2-miRNA1) influences biogenesis and activity of other secondary miRNAs (miRNA 2 in red) that share the binding sites on the same 3′ UTR of common target mRNA in a cooperative manner. The miRNP2 (Ago2-miRNA2) targets new target mRNA B (green) that do not have miRNA 1 target sites. In step c, increased coordinated biogenesis of primary and secondary miRNAs leads to increased target mRNA degradation or translational repression of both primary and secondary target mRNAs (mRNA A and B).

CB regulator-target relationships can be readily predicted based on the assumptions that we have utilized in this study. Based on the data collected during this work, we developed a repository that can be queried to obtain a set of miRNAs likely to exhibit coordinate biogenesis in a particular scenario, since miRNA and mRNA expression data are utilized to infer the most likely relationships. It is plausible that 302 miRNAs are likely to exhibit the CB phenomenon in murine cells and that 1,433 miRNAs are likely to exhibit the CB phenomenon in human cells. Utilizing these miRNAs and expression profiles as the query, one can obtain high-confidence CB pairs wherein the CB regulator and secondary effector miRNAs are upregulated and the corresponding target mRNAs are downregulated or vice versa. Alternately, a list of low-confidence CB pairs is calculated considering that CB regulator-target miRNAs and mRNAs are all expressed significantly (Fig. 11A).

### Progressive dampening mode of action of primary miRNAs on secondary to tertiary miRNAs and their targets.

How does coordinated miRNA biogenesis affect the widespread gene regulation observed in higher eukaryotes? When we plotted the concentration of miR-125b, the secondary miRNA, against the changes in levels of primary miR-146a-5p in RAW264.7 cells, we found a curve connecting the data which can be represented by an equation relating changes in the miR-125b level with the changes in the miR-146a-5p concentration (Fig. 11B). It can be hypothesized for any secondary miRNA (with a lower number of binding sites on the target mRNA) that the changes in its expression are primarily controlled by primary miRNA (with a higher number of binding sites on the same target mRNAs) and the concentration of common target mRNA that they both target. We have extrapolated this concept further to suggest that the concentration of the miRNA in the *n*th layer in this regulatory circuit is influenced by the concentration of miRNA in the (*n* − 1)th layer and the mRNA having binding sites for the *n*th and (*n* − 1)th layer miRNAs. This assumption led us to formulate the equation to predict how the concentration change of individual miRNAs affects the primary and secondary mRNAs (Fig. 11C). It can be assumed that any mRNAs having binding sites for specific sets of miRNAs will be affected not only by the miRNA in the same regulatory layer (i.e., in the *n*th layer) but also by the concentration of miRNAs in the previous layers (*n* − 1, *n* − 2, … etc.) that are indirectly influencing the concentration of the miRNA at the same (*n*th) level through our proposed coordinated biogenesis phenomenon (Fig. 11D). The distance between two layers (*n* and *n − x*, where *x* = 1, 2, 3…) determines the effect of an miRNA in a particular layer on the mRNA targets in another layer, and the repressive effect should decrease gradually with the value of *x*. Therefore, the expression of an mRNA is influenced by sum of the effects of individual miRNAs present in different layers that do not require one canonical binding site on that mRNA. Thus, the miRNA repository of a cell is influenced by the status of multiple miRNAs and the network they form, which have a cumulative effect on the mRNA transcriptome of the cell (Fig. 11C and D). A disease condition that affects a specific primary miRNA pool thus could be connected with a changed expression of the “secondary” target genes, which are without the binding sites for the primary miRNAs but are differentially expressed prominently in the disease condition due to changed levels in the secondary or tertiary miRNAs.

## DISCUSSION

Here, we report the target-dependent coordinated biogenesis (TDCB) of miRNAs, where target mRNAs increased the biogenesis of a family of secondary miRNAs that share binding sites in the 3′ UTR of common target mRNAs having binding sites for a primary miRNA. We identified and unraveled how the TDCB operates in a physiological context to regulate the miRNA network and fine-tune the inflammatory responses. Previous works have been focused on the regulation of a single miRNA activity by its targets, while our findings emphasize an understanding of the sequential molecular events in a physiological context that drives a cascade of expression of miRNAs affecting their expression at the posttranscriptional level. Earlier, PDCD4-mediated coregulation of miR-21 and miR-499 has been documented, where activity and stabilization of one miRNA species was shown to influence others (50). We have proposed a coordinated biogenesis model of miRNA in which the presence of multiple target sites of a “primary” miRNA increases the fall-off rate of Dicer1 molecules from the miRISC for its reassociation with new Ago2 to form a new miRISC with secondary miRNAs and favor their association of shared 3′ UTR. This, in turn, increases the biogenesis of not only “primary” miRNA but also of other secondary miRNAs having adjacent target sites on the coregulated mRNAs in a coordinated manner. This results in the repression of secondary mRNAs targeted by these newly generated secondary miRNAs but not by the primary miRNAs (Fig. 11E). These findings broaden our basic understanding from a “one miRNA to one mRNA” regulation to an miRNA-targetome-based coordinated gene repression network in the field of miRNA-mediated gene regulation, where cooperativity among target sites influences miRNA biogenesis.

We hypothesized that TDCB is a general and complex phenomenon probably occurring in every miRNA-driven pathway with varied layer of complexity, but it was challenging to single out the phenomenon due to the existence of subtle cross talk of different interlinked pathways inside the cell. We were looking for a well-conserved miRNA-responsive pathway where miRNAs act prominently to regulate multiple pathways, making it a suitable candidate to showcase TDCB. Expressions of many endotoxin-responsive miRNAs are mostly controlled in a NF-κB-dependent manner, and their mode of action varies as they form reciprocal regulatory relationships as well. For example, miR-155 and miR-146a-5p have been proven to be the critical reciprocal mediators of this phenomenon (51, 52). NF-κB binds to the putative promoter elements of many endotoxin-responsive miRNAs in a time-dependent manner that diversifies the responses into immediate early, early, and later immune responses (14, 20). This led us to speculate that sensing mechanisms may exist among miRNAs that coordinate the diversified network to balance the immune responses inside the macrophages. This intrigues us to unravel the molecular mediators of the phenomenon. The absence of a prominent anti-inflammatory miRNA, miR-146a-5p, on LPS-induced macrophages shows differential expression of LPS-responsive miRNAs that share target sites with the miR-146a-5p targetome. To broaden our observation, whole-cell transcriptomics data also suggest that a group of miRNAs also exhibits miR-146a-5p-mediated CB, irrespective of predocumented LPS-induced behavior. An integrated biochemical and computational approach has been designed to predict and validate probable regulatory relationships between miRNAs in which one miRNA is likely to influence the biogenesis of the other. As per our hypothesis, a CB regulator (miRNA-1) is upregulated to induce the biogenesis of another miRNA (miRNA-2) by coordinated action, while the corresponding targets (mRNA-A) with binding sites for both miRNAs are likely to be downregulated. Further, as a result of this phenomenon, a corresponding downstream effect on a class of secondary mRNA targets (mRNA-B) containing the binding sites for the secondary effector miRNA-2 should be observed. In order to study this mechanism, we utilized miRNA-mRNA regulatory interaction data to predict a set of miRNAs that are likely to act as CB regulators and influence the biogenesis of another miRNA target. Thus, a probable miRNA biogenesis regulatory or meta-interaction network has been predicted in mice and in humans. Utilizing large-scale analysis of expression data of monocytes exposed to LPS, we could identify a number of miRNAs which are likely to be regulated by miR-146a-5p. miRNAs such as miR-16-5p, miR-26a-5p, miR-27b-3p, miR-30e-5p, miR-93-5p, and miR-98-5p are among those which have been identified as probable secondary miRNAs in the miR-146a-5p regulatory network of coordinate biogenesis in mice and in humans. Moreover, the predicted CB relationships (miRNA-1:mRNA-A to miRNA-2:mRNA-B) for mmu-miR-146a-5p have been studied in detail, and we could validate that miR-146a-5p likely regulates the biogenesis of miR-16-5p and miR-21-3p that in turn regulates the expression of secondary effector mRNA as well. By studying the miRNA binding site locations and numbers, we could predict possible CB pairs and their network of secondary effector miRNAs and mRNAs. However, there are subtle differences between the predicted and experimentally analyzed data sets that could be due to the lack of granularity of the sequencing data, where all binding sites may not have been characterized. Further, the prevalences (counts) of different miRNA pairings (CB relationship pairs) that occurred across all genes A were determined. Some of these relationships along with others that had literature support were studied in detail. A substantial number was studied to establish our proposed hypothesis. We also utilized other large-scale expression analysis data sets to support the TDCB paradigm. miR-146a-5p was identified as exhibiting this coordinate biogenesis phenomenon to regulate the macrophage immune response upon stimulation via LPS.

Different endotoxin-responsive signaling modulators of NF-κB, MAPK, and Akt1 signaling pathways are there and endotoxin-responsive miRNAs interact among each other to create cross talk to elicit the cascaded immune responses. Hence, to negate the influence of endotoxin-responsive molecules on miR-146a-5p, we adopted an LPS-free miR-146a-5p expression system that precisely induces miR-146a-5p expression in macrophages and monitored the aftereffect related to coordinated miRNA biogenesis and function. Biochemical analysis and RNA sequencing data corroborated each other and also strengthened and revalidated our observations for activated macrophages.

On a precise note, these mechanisms probably have finer levels of cooperative regulation vested upon multiple comediators which may not be exclusively controlled by groups of the TDCB class of miRNAs. Rather, the integrative network formed by the “miRNA” and “effector target mRNAs/proteins regulated by miRNA” imparts the highest level of cooperation to elicit this response. Ultimately, this interconnected complex network gives rise to a signaling web in which CB is probably eliciting the phenotypes. For instance, the regulation of TNF-α expression might be controlled at different levels by different modulators. IRAK1/TRAF6-controlled phosphorylation of p38 and ERKs activates different MAPK-activated protein kinases to further activate different transcription factors, production of proinflammatory cytokines, and cell surface receptors that may ultimately lead to the onset of cellular apoptosis (53–55). The increased production of TNF-α has a huge impact on iNOS synthesis as well (56, 57). TNF-α also bears binding sites for miR-125b (58). Probably, this layering of cascaded regulation is manifested in the temporal expression of TNF-α. In contrast, as another example, miR-21, another important CB candidate, has a significant repressive effect on p38‐CHOP and JNK signaling to inhibit the proinflammatory phenotype and macrophage apoptosis modulated by MAP2K3 expression (59). PDCD4, another proinflammatory protein, is also suppressed by miR-21, which leads to decreased NF-κB activity and increased production of anti-inflammatory cytokine IL-10 (23). We could also observe significant derepression of these molecules (i.e., MAP2K3 and PDCD4) under miR-146a-5p-inhibited conditions, where neither of these mRNAs bears sites for miR-146a-5p. This probable regulatory network axis mediated by “miR-146a-5p→TRAF6→miR-21→PDCD4/MAP2K3/PTEN→p38/NF-κB/JNK” might be one among many that exist in activated macrophages. These findings led us to predict a coordinated biogenesis-regulated inflammatory network (CBIN) that probably exists in cells post-endotoxin treatment. Other multilinear coregulations have also been reported in the context of inflammation that even further support our TDCB-based interactive analysis for fine-tuning each and every orchestrated response (60, 61). Considering all this together, we could see that groups of familiar miRNAs act in coordination but in a target-dependent manner to protect macrophages through proinflammatory cytokine silencing and inhibition of apoptotic pathways, resulting in tissue healing.

In addition, we have also determined the likely conditions under which this CB phenomenon can occur and developed a computational module to predict regulatory miRNAs and their network under these contexts. Analysis of a few CB regulatory networks suggests that multiple miRNAs may potentially exhibit this phenomenon in a condition-specific, cell type-specific, and species-specific manner. There might also be other different factors which influence our observations. Stabilization and turnover of miRNA, strength of miRNA-target site pairing, distance between cooperative sites, or influences of other RNA binding proteins may prove to be crucial for this type of cooperation. Not only Dicer1 processivity or complementary seed sequence pairing but also spatial binding among molecules might impart a crucial role for this type of interaction. Further experiments on multiple various aspects may give a better understanding of the structural basis of this phenomenon. We have observed no or minimal contribution of transcription in the overall increase of secondary miRNAs in the TDCB process. Overall, we have unraveled a unique phenomenon in which the TDCB of miRNAs contributes in building a molecular framework to balance the immune responses in activated macrophages.

## MATERIALS AND METHODS

### Cell culture and reagents.

RAW264.7 cells were cultured in RPMI 1640 medium (Gibco) supplemented with 2 mM l‐glutamine, 0.5% β‐mercaptoethanol, and 10% heat‐inactivated fetal calf serum (FCS) (or 10% Tet-system approved FCS for experiments related to tetracycline-inducible expression construct system). Primary murine peritoneal exudate macrophages (PEC) were boosted by 4% starch injected intraperitoneally. After 72 h of starch-mediated stimulation, the mice were sacrificed, and peritoneal macrophages were collected after washing and clearing of the peritoneal cavity with cold 1× phosphate-buffered saline (PBS). Cells were spun down and grown in RPMI 1640 supplemented with 2 mM l-glutamine and 10% heat-inactivated FCS.

### Expression plasmids, transfection, and treatment of cells.

Pre-miR-122 or pre-miR-146a sequences were cloned within pTRE-Tight-BI vector (Clontech) in NheI and NotI sites or Bam HI and HindIII to make induced miR-122 (imiR-122) and induced miR-146a (imiR-146a) constructs, respectively. pTet-On advanced vector (Clontech) expressing Tet-responsive reverse transactivator was used to activate the Tet-On system inside the cells. Plasmid expressing green fluorescent protein (GFP) was amplified and cloned in pCI-neo vector to make GFP-Con. Precursor miRNA-125b sequence cloned into pU61 Hygro (Genscript, USA) vector and MAD1-3′UTR were cloned into the linearized pMIR-REPORT vector (Applied Biosystems, Foster City, CA, USA) downstream of the Luc gene to make pmiR-125b and pRL-MAD1-3′UTR respectively, which were kind gifts from Susanta Roy Chowdhury (30). FLAG- and HA-tagged human Ago2 expression plasmid FHA-Ago2 was a kind gift from Gunter Meister (62). Ago3 and Ago4 expression plasmids were from Witold Filipowicz. The human IRAK1-3′UTR was cloned downstream of the pRL-con vector using the XbaI and NotI restriction sites to make the pRL-IRAK1-3′UTR construct. Downstream of the pRL-con plasmid, the 3′ UTR of the HMGA2 gene with intact let-7a binding sites was cloned (RL-HMGA2 3'UTR), a kind gift from Anindya Dutta (63). Downstream of the pRL-con plasmid, respective sequences were cloned between the XbaI and NotI restriction sites for pRL-3xb-miR-146a, pRL-3xb-miR-146a-1xb-miR-125b, pRL-1x-perf-miR-125b, and pRL-1x-perf-miR-142-3p constructs.

Lipofectamine 2000 (Life Technologies) was used as a transfection reagent for this study per the manufacturer’s protocol. A 30 nM concentration of Ambion anti-miR miRNA inhibitor, either anti-miR-146a-5p (AM10722), anti-miR-155-5p (AM13058), anti-miR-125b (AM10148), miR-142-3p (AM10398), or anti-miR-122-5p as a control (AM11012), was transfected in RAW264.7 cells for anti-miR experiments. The macrophages were induced with 10 ng/mL Escherichia coli O111:B4 LPS (Calbiochem, La Jolla, CA) for activation unless specified otherwise. A 10 μg/mL final concentration of α-amanitin was used for transcriptional blockage experiments (Calbiochem). For the immunoprecipitation study, 1.5 μg of FLAG-HA-Ago2 (or other different HA-tagged Ago variants) expressing plasmid was transfected along with anti-miRs in a 6-well cell culture plate format. The transfection method used here has followed the manufacturer’s protocol. siDicer1, specific for mice, was transfected at a 30 nM concentration in RAW264.7 cells. Dharmacon SMARTpool ON-TARGETplus siRNAs against Dicer1 were bought from Thermo Scientific. siCon (ON-TARGETplus nontargeting pool) was used as a control. Transfection of siRNAs was carried out using RNAiMax (Life Technologies) per the manufacturer’s protocol. For experiments related to inducible expression systems on RAW264.7 cell, 600 ng of Tet-On plasmid and 400 ng of inducible miR-146a-5p expression plasmid were transfected in 12-well cell culture formats. FuGENE HD (Promega) transfection reagent was used for this per the manufacturer’s protocol.

### Luciferase assay.

Luciferase reporter assays were carried out using a dual-luciferase assay kit (Promega), by following the manufacturer’s instructions, on a Victor X3 plate reader (PerkinElmer). *Renilla* luciferase (RL) luminescence was normalized with firefly luciferase (FF) activities to calculate fold repression. Firefly-normalized RL values were plotted. For the luciferase reporter study in RAW264.7 cells, 200 ng firefly luciferase (FL), along with 40 ng of *Renilla* luciferase (RL) reporter, was co-transfected along with the necessary reporter plasmid or anti-miRs in a 24-well cell culture format. Fold repression is represented as the ratio of firefly-normalized RL activity for RL-con to the RL-experimental condition (fold repression = RL-con/RL-experimental).

### Animal ethics.

The animal facility of the CSIR-Indian Institute of Chemical Biology provided all the required adult BALB/c mice (of any sex), and we followed the National Regulatory Guidelines issued by the Committee for the Purpose of Supervision of Experiments on Animals, Ministry of Environment and Forest, Government of India, for all experiments performed.

### Isolation of RNAs and real-time PCR quantification of mRNA and miRNAs.

TRIzol reagent (Life Technologies) was used for isolation of RNA at the total cellular level or from organelle fractions. Isolation of small RNAs was performed using a small-RNA-specific RNA isolation kit (mirVana miRNA isolation kit; Invitrogen catalog no. AM1561) that would specifically isolate the RNA species of ≤200 bases. For mRNA quantification, random nonamers (Eurogentec reverse transcriptase core kit) was used to prepare cDNA by using 200 ng of the total RNA for 10 μL of reaction mixture, and the produced cDNA was used for comparative quantitation of mRNA expression. Real-time (reverse transcriptase) PCR from cDNA was done with the Mesa Green qPCR Mastermix Plus for SYBR assay-low ROX (Eurogentec). For real-time-based quantification to study comparative differential expression, the following primers were used: TRAF6 (forward, 5′-CCTCAAGATGTCTCAGTTCCATC-3′; reverse, 5′-GTTCTGCAAAGCCTGCATC-3′), IRAK1 (forward, 5′-CTGTGGCCCTGGATCAAC-3′; reverse, 5′-GAAAAGCTGGGGAGAGGAAG-3′), HIF1AN (forward, 5′-AGTGCCAGCACCCATAAGTTC-3′; reverse, 5′-AACCCAAGAAG TCCATGACAATC-3′), MAP2K7 (forward, 5’GATCCCACCAAGCCTGACTATG3′; reverse, 5′-ACTGGAAGTCCCCTGAGAAGCC-3′), HMGB1(forward, 5′-GGCCTTCTTCTTGTTCTGTT-3′; reverse, 5’GCAACATCACCAATGGATAA-3′), ADCY9 (forward, 5′-GCAAAATGGCTGTCAA GACGAGC-3′; reverse, 5′-CTGGCTGTTAGTGAGCTTCTCC-3′), PDCD4 (forward, 5′-GTAGATTGTGTACAGGCTCGAG-3′; reverse, 5′-CCCACACACTGTCTTTCCGC-3′), MAP2K3 (forward, 5′-GAGGCTGATGACTTGGTGAC-3’; reverse, 5′-GCACCATAGAAGGTGACAGTG-3′), pre-miR-155 (forward, 5′-CTGTTAATGCTAATTGTGATAGG-3′; reverse,5′-CTGTTAATGCTAACAGGTAGG-3′), pre-miR-146a (forward, 5′-AGCTCTGAGAACTGAATTCC-3′; reverse, 5′-GCTGAAGAACTGAATTTCACAG-3′), pre-miR-21 (forward, 5′-TGTCGGATAGCTTATCAGACTG-3′; reverse, 5′-TGTCAGACAGCCCATCG-3′), pre-miR-125b (forward, 5′-AGTCCCTGAGACCCTAACTTG-3′; reverse, 5′-AGCTCCCAAGAGCCTAAC-3′), pre-miR-142 (forward, 5′-ACCCATAAAGTAGAAAGCAC-3′; reverse, 5′-CATCCATAAAGTAGGAAACAC-3′), pri-miR-21 (forward, 5′-CCAGAGATGTTTGCTTTGCTTTA-3′; reverse, 5′-CCATGATGCTGGGTAATGTTTG-3′), pri-miR-125b (forward, 5′-ACGAGTCTGCAACCGAAAT-3′; reverse, CTCTGTGTCTCTCTGTCTCTCT-3′), pri-miR-142 (forward, 5′-AAAGCAGGTGGCCTGAAGAA-3′; reverse, 5′-AGATGCTCACCTGTTTCCTTGAT-3′), Mis18bp1 (forward, 5′-TCTTCCAAAGCACAAACCTGG-3′; reverse, 5′-TTCCCACTTTGGCAGTTATC-3′), MCM 5 (forward, 5′-ATCCAGGTCATGCTCAAGTC-3′, reverse, 5′-TTCCTGGGAAGGGCATAGC-3′), NCAPG2 (forward, 5′-ACTAGATGAATTATCAAGGAAAC-3′; reverse, 5′-CATTTATT ATAGATACAGAAGCAAG-3′), RRM2 (forward, 5′-TTTCTATGGCTTCCAAATTGC-3′; reverse, 5′-GATGCAAAAGAACCGGAAAAG-3′), ASPM (forward, 5′-ATATTAACCCCTGATGACTTC-3′; reverse, 5′-AACCATTTTTTCAGAAGTAAAC-3′), NCAPD3 (forward, 5′-AAGGCTTTTCATATCTGGTCC-3′; reverse, 5′-TTGGGAAGATGCTTTGCAATATG-3′), FEN1 (forward, 5′-AACACAATGATGAGTGCAAAC-3′; reverse, 5′-ATGCACAGATCCACAAACTG-3′), TNF-α (forward, 5′-GTCTCAGCCTCTTCTCATTCC-3′; reverse, 5′-TCCACTTGGTGGTTTGCTA-3′), IL-1β (forward, 5′-GACCTTCCAGGATGAGGACA-3′; reverse, 5′-CCTTGTACAAAGCTCATGGAG-3′). 18S rRNA (forward, 5′-TGACTCTAGATAACCTCGGG-3′; reverse, 5′-GACTCATTCCAATTACAGGG-3′) or GAPDH (forward, 5′-CAGGGGGGAGCCAAAAGGG-3′; reverse, 5′-CTTGGCCAGGGGTGCTAAGC-3′) was used as an endogenous control. The comparative threshold cycle (*C_T_*) method was used for relative quantitation that typically uses normalization by the housekeeping mRNA expression, namely, 18s rRNA or GAPDH mRNA levels. For the ChIP assay, the following primers were used: HIF1AN (forward, 5′-GGAACGGGAAGTGTGTAGTAAG-3′; reverse, 5′-AACCCAAGAAGTCCATGACAATC-3′), MAP2K7 (forward, 5′-GGTGGAGAGGTGTGTTAGAAAG-3′; reverse, 5′-GACATTCCTTGAAGAGCTAGGG-3′), HMGB1 (forward, 5′-CAAGCAGCCCTATGAGAAGAA-3′; reverse, 5′-ACTATTGGCCGTTCCTGTATG-3′), and ADCY9 (forward, 5′-GTGATGACCTCCCAACCATAAA-3′; reverse, 5′-TGGAAACAGGGAGTGAGTAATG-3′).

Cellular or organellar miRNA levels were quantified using a TaqMan-based miRNA-specific assay kit starting with 50 ng of total RNA. The reverse transcription mix was used for primary PCR amplification of miRNAs, followed by TaqMan Universal PCR Master Mix No AmpErase (Applied Biosystems). The samples were analyzed in triplicate for each biological replicate. The comparative *C_T_* method was used in cases where the candidate miRNA was normalized with endogenous control U6 snRNA. The candidate miRNA-specific TaqMan MicroRNA Assays (Applied Biosystems) used were as follows: for let-7a, assay ID 000377; miR-122, assay ID 000445; miR-146a, assay ID 000468; miR-155, assay ID 002571; miR-21, assay ID 000397; miR-125b, assay ID 000449; miR-142-3p, assay ID 000464; miR-16, assay ID 000391; miR-21-3p, assay ID 002493; and U6 snRNA, assay ID 001973. All reactions were performed in an Applied Biosystems 7500 real-time system or a Bio-Rad CFX96 real-time system. Cycles were set as described in the manufacturer’s protocol.

### Immunoprecipitation assay.

The procedure for immunoprecipitation (IP) of Ago2 was performed in accordance with published protocols (64, 65). FLAG-M2 agarose beads (Sigma) were washed three times with 1 × IP buffer (20 mM Tris–HCl, pH 7.5, 150 mM KCl, 1 mM MgCl_2_). Cells were lysed in lysis buffer (20 mM Tris-HCl, pH 7.4, 200 mM KCl, 5 mM MgCl_2_, 1 mM dithiothreitol [DTT], 1 × EDTA-free protease inhibitor [Roche], 5 mM vanadyl ribonucleoside complex [Sigma], 0.5% Triton X-100, 0.5% sodium deoxycholate) at 4°C for 30 min, followed by 10 s of sonication, three times, with a 30 s incubation on ice in between. Lysate was centrifuged at 16,000 × *g* for 5 min at 4°C after lysis. Immunoprecipitation was carried out for 16 h at 4°C. After washing the beads with 1× IP buffer, the beads were divided in two halfs for protein and RNA expression analysis. One half was subjected to RNA isolation with TRIzol LS and another part to Western blotting with 1 × SDS sample buffer.

### ChIP analyses.

The ChIP assay was performed as described previously (66). The eluted DNA was used for the subsequent quantitative PCR (qPCR) analyses (Bio-Rad CFX96TM real-time system) for quantifying the amount of DNA. IgGs corresponding to the target antibodies (RNA Pol II) were used as a control as mentioned.

### Western blotting.

Western blotting of proteins was performed as described in published protocols (18). The cell lysates or immunoprecipitated proteins were analyzed by SDS-PAGE. After completion of electrophoresis, the proteins were transferred to a polyvinylidene fluoride or polyvinylidene difluoride (PVDF) membrane, followed by blocking at 4°C for a minimum of 1 h. The blots were probed overnight at 4°C with the primary antibodies. The antibodies used were as follows: mouse anti-Ago2 (Abnova), 1:1,000; rabbit anti-DICER1 (Bethyl), 1:8,000; rat anti-HA (Roche), 1:1,000; horseradish peroxidase (HRP)-conjugated anti-β-Actin (Sigma), 1:10000; rabbit anti-TRBP2 (Cell Signalling), 1:1000; rabbit anti-Drosha (Bethyl), 1:8,000; rabbit anti-P-p38 (Cell Signaling), 1:1,000; rabbit anti-P-ERK1/2 (Cell Signaling), 1:1,000; mouse anti-HSP70 (Santa Cruz Biotechnology), 1:1,000; rabbit anti-MSK1 (Cell Signaling), 1:1,000; mouse anti-HSP70 (Santa Cruz Biotechnology), 1:1,000; rabbit anti-cleaved PARP (Cell Signaling), 1:1,000; rabbit anti-cleaved caspase 9 (Cell Signaling), 1:1,000; and rabbit anti-P-Akt (Ser-473) (Cell Signaling), 1:1,000. Visualization of all Western blots was performed using an UVP BioImager 600 system equipped with VisionWorks Life Science software (UVP) V6.80. ImageJ software was used for densitometric analysis of blots for the relative quantification of bands.

### Measurement of NO.

Nitric oxide (NO) generation in response to LPS treatment post-anti-miR-146a transfection was quantified by the Griess reaction as described previously (67, 68). The amount of NO produced by the cell was measured from culture supernatants after construction of a standard curve with different concentrations of sodium nitrite. The concentration of accumulated NO was expressed as micromolar NO.

### Cytokine measurement by ELISA.

After completion of the necessary experiments, culture supernatant was collected. Using commercially available ELISA kits (R&D Systems), the amounts of TNF-α produced under specific conditions were measured according to the manufacturer’s protocol.

### TUNEL assay.

TUNEL assays were performed using a DeadEnd fluorometric TUNEL system kit (Promega) per the manufacturer’s protocol. The protocol was described previously (69). The percentage of apoptotic cells was counted by analyzing multiple microscopic fields that are represented on a bar graph.

### Measurement of phagocytosis using fluorescent latex beads.

Red fluorescence-labeled latex beads (Fluorescent Red; Sigma-Aldrich) were diluted in RPMI 1640 medium and added to macrophages at a cell-to-bead ratio of 1:10 for 6 h. LPS (10 ng/mL) treatment was given in cases of anti-miR-146a or negative control transfections. Cells were fixed with 4% paraformaldehyde (PFA) and mounted in Vectashield without DAPI (4′,6-diamidino-2-phenylindole). The cell cytoskeleton was stained with phalloidin 488. Red latex bead phagocytosis was imaged in a Zeiss LSM800 confocal microscope. The percentage of phagocytosis was calculated from different captured microscopic fields.

### Prediction of coordinate biogenesis interaction relationship data.

Experimentally determined miRNA binding site information (genomic location, number of sites) was collected from TarBase (70) and miRTarBase (71) databases. Interactions (2,33,601) between human miRNA (2,588) and their target genes (8,808) from miRTarBase (both “strong” and “weak” interactions which had genomic binding positions listed) were considered. Similarly, 24,650 interactions were also retrieved between mouse miRNA (847) and their target genes (3,940). Additional interactions (2,82,181 and 1,34,997, respectively) were retrieved between human miRNA (1,026) and target genes (13,811) and between mouse miRNA (434) and genes (10,581) from the TarBase (“direct” interactions which had genomic binding positions listed were considered) (70). TarBase and miRTarBase data were combined and pooled to extract all possible experimentally verified miRNA-mRNA interactions in human and mouse, respectively.

Under certain conditions, miRNA can influence the biogenesis of other miRNAs, a phenomenon referred to as coordinate miRNA biogenesis. In order to computationally predict miRNAs likely to exhibit coordinate biogenesis, the CB regulator and its associated targets were identified according to the following conditions: for condition 1, miRNA-1 (e.g., miR-146a) should have 2 or more binding sites in tandem with miRNA-2 (with a fewer number of binding sites than miRNA-1) on the same 3′ UTR of the common target gene/mRNA (within any gene, say “gene A”); for condition 2, the target gene (“gene B”) of secondary effector miRNA (miRNA-2) should contain 2 or more binding sites of miRNA-2 and does not carry binding sites for miRNA-1 on its 3′ UTR.

These two conditions generated multiple triads, each of which includes miRNA-1, miRNA-2 (secondary effector), and gene B (secondary effector gene) (Fig. 4A). Further, the expression level of the secondary effector gene may indicate a change in miRNA-2 biogenesis (secondary effector) mediated by miRNA-1. miR-146a-5p is the miRNA-1 in our context, as we wanted to explore the probable cooperative effect of this miRNA.

This relationship of miRNA-1:mRNA-A → miRNA-2:mRNA-B was derived between miRNA-1 and miRNA-2 considering the binding site information data that were utilized to determine the coordinate biogenesis regulatory network for miR-146a-5p in Mus musculus (see Table S6 in the supplemental material) and Homo sapiens, respectively. Additionally, a prevalence count corresponding to different miRNA coordinate biogenesis pairs that occur across all genes A has been determined; this value can be utilized to assign precedence to more likely coordinate biogenesis pairs. Further, to determine a set of regulator-target relationships that we can validate or study in an experimental setup, we utilized large-scale expression analysis data sets to prune our regulator-target relationship list.

### Contextualization of coordinate biogenesis pairs for experimental validation.

Expression analysis data from murine macrophages and isolated human monocytes that were transformed into macrophages exposed to LPS (10 ng/mL) for 24 h (condition similar to that of experimental data sets considered in Fig. 2) were considered (GEO accession no. GSE19490 [72] and GSE85333 [73]). Differentially expressed mRNAs in each case were determined considering a fold change threshold of 1.5 and a *P* value threshold of 0.05. Further, these differential expression data were mapped in Homo sapiens and Mus musculus coordinate biogenesis regulator-target relationship data for miR-146a-5p according to the following conditions.

Regulator-target relationships in which the secondary effector gene (gene B) and the primary target for miR-146a (gene A) were both found to be downregulated, considering “miR-146a-mediated coordinated repression” was selected. The respective fold changes and *P* values have been included in Table S1. Moreover, coordinate biogenesis miRNA pairs occurring in genes where gene A has a direct regulatory relationship with gene B, e.g., where gene A could regulate gene B as a transcription factor, have been filtered out from the validation set. The filtering was performed to ensure that the effect on the secondary effector gene is most likely due to the increase in biogenesis of miRNA-2 as a result of the coordinate biogenesis relationship that they are likely to share.

### Validation of CB regulator-target pairs utilizing large-scale expression profiling.

Small-RNA sequencing analysis to determine differentially expressed miRNAs in the background of miR-146a-5p overexpression in RAW264.7 cells was performed to identify miRNAs whose expression is influenced when the CB regulator (miR-146a-5p) expression is upregulated. miRNAs with a fold change (FC) of >0 and a *P* value of ≤0.05 were considered upregulated, and miRNAs with a FC of <0 and a *P* value of ≤0.05 were considered downregulated. Since CB regulator expression is upregulated, the expression of another miRNA (miRNA-2) should be upregulated by coordinate biogenesis. Thus, by utilizing miRNA expression profiles, such CB regulator-target relationships for miR-146a-5p were identified. It was further studied whether these CB regulator-target relationships are widely observed in macrophages responding to LPS exposure wherein miR-146a-5p is likely to be overexpressed. Additionally, considering target genes of miR-146a-5p and secondary effector miRNA in the miR-146a-5p coordinate biogenesis network, a pathway overrepresentation analysis (74) was performed to determine the physiological relevance of this network. Further, similar CB regulator-target relationships were identified for other miRNAs likely to be overexpressed in macrophages as a result of LPS exposure, and these were further validated with the help of joint miRNA-mRNA expression profiling data.

### Determining the coordinate biogenesis regulatory network for miR-155-5p, miR-125a-3p, and miR-146a-5p.

The coordinate biogenesis relationship (miRNA-1:mRNA-A → miRNA-2:mRNA-B) was determined utilizing the conditions (conditions 1 and 2) mentioned above by considering mmu-miR-155-5p as miRNA-1 to determine the probable coordinate biogenesis regulatory network of miR-155-5p. This network was studied in detail by considering the mRNA expression profiles in murine macrophages (miR-155 knockout) responding to LPS (GEO accession no. GSE77425 [47]). Additionally, the role of miR-155 in macrophage polarization was also predicted by considering the miRNA-mRNA expression profiles in polarized and unpolarized murine macrophages (48). Subsequently, the coordinate biogenesis regulatory network of miR-125a-3p was also determined by considering mmu-miR-125a-3p as miRNA-1. Further, the probable role of miR-125a-3p as a CB regulator was studied in macrophage polarization (48) and hematopoietic stem cell differentiation (GSE33691 [49]). Moreover, miR-146a-5p coordinate biogenesis networks in murine monocytes and human monocytes under different LPS exposure conditions were determined utilizing miRNA expression profiling data sets (GSE87396 [75], GSE125572 [69]), while the has-miR-146a-5p CB regulatory network in Mycobacterium tuberculosis-infected macrophages was determined by considering a previous study (76).

### Web server for determining CB regulator-target relationships.

The coordinate biogenesis data for each CB regulator (miRNA) can be obtained from the COORD-BIO repository. Herein, the data mined during this work to study the coordinate biogenesis phenomenon are queried by considering a particular CB regulator and expression profiles (miRNA and mRNA) in a scenario of interest. The front end of the server is HTML, PHP, and Java based, while a perl script determines all possible CB regulator-target relationships for a particular CB regulator (miRNA) based on the conditions proposed for the coordinate biogenesis phenomenon to occur. Further, context-specific miRNA and mRNA expression profiling data may be utilized to determine the most likely sets of CB relationships. The web server for determining probable CB miRNA pairs and associated secondary effector mRNA is freely available for use (http://www.hpppi.iicb.res.in/coordB2/index.html).

### Data availability.

miRNA sequencing data associated with this study are freely available in GEO. The data discussed in this publication have been deposited in the NCBI Gene Expression Omnibus (77, 78) and are accessible under GEO accession number GSE172473. KEGG analysis was done as per the published protocol (79). Differential mRNA expression analysis was done as per protocol published earlier (80, 81).

## References

[1] O'Brien J, Hayder H, Zayed Y, Peng C. 2018. Overview of microRNA biogenesis, mechanisms of actions, and circulation. Front Endocrinol (Lausanne) 9:402. 10.3389/fendo.2018.00402. 30123182 PMC6085463

[2] Bartel DP. 2004. MicroRNAs: genomics, biogenesis, mechanism, and function. Cell 116:281–297. 10.1016/s0092-8674(04)00045-5. 14744438

[3] Bose M, Bhattacharyya SN. 2016. Target-dependent biogenesis of cognate microRNAs in human cells. Nat Commun 7:12200. 10.1038/ncomms12200. 27448149 PMC4961841

[4] O'Connell RM, Rao DS, Chaudhuri AA, Baltimore D. 2010. Physiological and pathological roles for microRNAs in the immune system. Nat Rev Immunol 10:111–122. 10.1038/nri2708. 20098459

[5] Chan JJ, Tay Y. 2018. Noncoding RNA:RNA regulatory networks in cancer. Int J Mol Sci 19:1310. 10.3390/ijms19051310. PMC5983611 29702599

[6] Salmena L, Poliseno L, Tay Y, Kats L, Pandolfi PP. 2011. A ceRNA hypothesis: the Rosetta Stone of a hidden RNA language? Cell 146:353–358. 10.1016/j.cell.2011.07.014. 21802130 PMC3235919

[7] Tay Y, Kats L, Salmena L, Weiss D, Tan SM, Ala U, Karreth F, Poliseno L, Provero P, Di Cunto F, Lieberman J, Rigoutsos I, Pandolfi PP. 2011. Coding-independent regulation of the tumor suppressor PTEN by competing endogenous mRNAs. Cell 147:344–357. 10.1016/j.cell.2011.09.029. 22000013 PMC3235920

[8] Poliseno L, Salmena L, Zhang J, Carver B, Haveman WJ, Pandolfi PP. 2010. A coding-independent function of gene and pseudogene mRNAs regulates tumour biology. Nature 465:1033–1038. 10.1038/nature09144. 20577206 PMC3206313

[9] Luna JM, Scheel TK, Danino T, Shaw KS, Mele A, Fak JJ, Nishiuchi E, Takacs CN, Catanese MT, de Jong YP, Jacobson IM, Rice CM, Darnell RB. 2015. Hepatitis C virus RNA functionally sequesters miR-122. Cell 160:1099–1110. 10.1016/j.cell.2015.02.025. 25768906 PMC4386883

[10] Denzler R, McGeary SE, Title AC, Agarwal V, Bartel DP, Stoffel M. 2016. Impact of microRNA levels, target-site complementarity, and cooperativity on competing endogenous RNA-regulated gene expression. Mol Cell 64:565–579. 10.1016/j.molcel.2016.09.027. 27871486 PMC5101187

[11] Kleaveland B, Shi CY, Stefano J, Bartel DP. 2018. A network of noncoding regulatory RNAs acts in the mammalian brain. Cell 174:350–362.e317. 10.1016/j.cell.2018.05.022. 29887379 PMC6559361

[12] Bose M, Barman B, Goswami A, Bhattacharyya SN. 2017. Spatiotemporal uncoupling of microRNA-mediated translational repression and target RNA degradation controls microRNP recycling in mammalian cells. Mol Cell Biol 37:e00464-16. 10.1128/MCB.00464-16. 27895152 PMC5288578

[13] Taganov KD, Boldin MP, Chang KJ, Baltimore D. 2006. NF-kappaB-dependent induction of microRNA miR-146, an inhibitor targeted to signaling proteins of innate immune responses. Proc Natl Acad Sci USA 103:12481–12486. 10.1073/pnas.0605298103. 16885212 PMC1567904

[14] O'Neill LA, Sheedy FJ, McCoy CE. 2011. MicroRNAs: the fine-tuners of Toll-like receptor signalling. Nat Rev Immunol 11:163–175. 10.1038/nri2957. 21331081

[15] Momen-Heravi F, Bala S. 2018. miRNA regulation of innate immunity. J Leukoc Biol 103:1205–1217. 10.1002/JLB.3MIR1117-459R. 29656417

[16] Nejad C, Stunden HJ, Gantier MP. 2018. A guide to miRNAs in inflammation and innate immune responses. FEBS J 285:3695–3716. 10.1111/febs.14482. 29688631

[17] Lu YC, Yeh WC, Ohashi PS. 2008. LPS/TLR4 signal transduction pathway. Cytokine 42:145–151. 10.1016/j.cyto.2008.01.006. 18304834

[18] Mazumder A, Bose M, Chakraborty A, Chakrabarti S, Bhattacharyya SN. 2013. A transient reversal of miRNA-mediated repression controls macrophage activation. EMBO Rep 14:1008–1016. 10.1038/embor.2013.149. 24030283 PMC3851954

[19] Goswami A, Mukherjee K, Mazumder A, Ganguly S, Mukherjee I, Chakrabarti S, Roy S, Sundar S, Chattopadhyay K, Bhattacharyya SN. 2020. MicroRNA exporter HuR clears the internalized pathogens by promoting pro-inflammatory response in infected macrophages. EMBO Mol Med 12:e11011. 10.15252/emmm.201911011. 32031337 PMC7059013

[20] Zhou R, Hu G, Gong AY, Chen XM. 2010. Binding of NF-kappaB p65 subunit to the promoter elements is involved in LPS-induced transactivation of miRNA genes in human biliary epithelial cells. Nucleic Acids Res 38:3222–3232. 10.1093/nar/gkq056. 20144951 PMC2879527

[21] Quinn SR, O'Neill LA. 2011. A trio of microRNAs that control Toll-like receptor signalling. Int Immunol 23:421–425. 10.1093/intimm/dxr034. 21652514

[22] Kurowska-Stolarska M, Alivernini S, Ballantine LE, Asquith DL, Millar NL, Gilchrist DS, Reilly J, Ierna M, Fraser AR, Stolarski B, McSharry C, Hueber AJ, Baxter D, Hunter J, Gay S, Liew FY, McInnes IB. 2011. MicroRNA-155 as a proinflammatory regulator in clinical and experimental arthritis. Proc Natl Acad Sci USA 108:11193–11198. 10.1073/pnas.1019536108. 21690378 PMC3131377

[23] Sheedy FJ, Palsson-McDermott E, Hennessy EJ, Martin C, O'Leary JJ, Ruan Q, Johnson DS, Chen Y, O'Neill LA. 2010. Negative regulation of TLR4 via targeting of the proinflammatory tumor suppressor PDCD4 by the microRNA miR-21. Nat Immunol 11:141–147. 10.1038/ni.1828. 19946272

[24] Murphy AJ, Guyre PM, Pioli PA. 2010. Estradiol suppresses NF-kappa B activation through coordinated regulation of let-7a and miR-125b in primary human macrophages. J Immunol 184:5029–5037. 10.4049/jimmunol.0903463. 20351193 PMC2882792

[25] Sun Y, Varambally S, Maher CA, Cao Q, Chockley P, Toubai T, Malter C, Nieves E, Tawara I, Wang Y, Ward PA, Chinnaiyan A, Reddy P. 2011. Targeting of microRNA-142-3p in dendritic cells regulates endotoxin-induced mortality. Blood 117:6172–6183. 10.1182/blood-2010-12-325647. 21474672 PMC3122940

[26] Hou J, Wang P, Lin L, Liu X, Ma F, An H, Wang Z, Cao X. 2009. MicroRNA-146a feedback inhibits RIG-I-dependent type I IFN production in macrophages by targeting TRAF6, IRAK1, and IRAK2. J Immunol 183:2150–2158. 10.4049/jimmunol.0900707. 19596990

[27] Agarwal V, Bell GW, Nam JW, Bartel DP. 2015. Predicting effective microRNA target sites in mammalian mRNAs. Elife 4:e05005. 10.7554/eLife.05005. PMC4532895 26267216

[28] Wang X, Ha T, Zou J, Ren D, Liu L, Zhang X, Kalbfleisch J, Gao X, Williams D, Li C. 2014. MicroRNA-125b protects against myocardial ischaemia/reperfusion injury via targeting p53-mediated apoptotic signalling and TRAF6. Cardiovasc Res 102:385–395. 10.1093/cvr/cvu044. 24576954 PMC4030511

[29] Nara K, Kawashima N, Noda S, Fujii M, Hashimoto K, Tazawa K, Okiji T. 2019. Anti-inflammatory roles of microRNA 21 in lipopolysaccharide-stimulated human dental pulp cells. J Cell Physiol 234:21331–21341. 10.1002/jcp.28737. 31042008

[30] Bhattacharjya S, Nath S, Ghose J, Maiti GP, Biswas N, Bandyopadhyay S, Panda CK, Bhattacharyya NP, Roychoudhury S. 2013. miR-125b promotes cell death by targeting spindle assembly checkpoint gene MAD1 and modulating mitotic progression. Cell Death Differ 20:430–442. 10.1038/cdd.2012.135. 23099851 PMC3572219

[31] Chendrimada TP, Gregory RI, Kumaraswamy E, Norman J, Cooch N, Nishikura K, Shiekhattar R. 2005. TRBP recruits the Dicer complex to Ago2 for microRNA processing and gene silencing. Nature 436:740–744. 10.1038/nature03868. 15973356 PMC2944926

[32] He F, Xiao Z, Yao H, Li S, Feng M, Wang W, Liu Z, Liu Z, Wu J. 2019. The protective role of microRNA-21 against coxsackievirus B3 infection through targeting the MAP2K3/P38 MAPK signaling pathway. J Transl Med 17:335. 10.1186/s12967-019-2077-y. 31585536 PMC6778380

[33] Zhang L, Ge Y, Fuchs E. 2014. miR-125b can enhance skin tumor initiation and promote malignant progression by repressing differentiation and prolonging cell survival. Genes Dev 28:2532–2546. 10.1101/gad.248377.114. 25403182 PMC4233245

[34] Chen S, Xue Y, Wu X, Le C, Bhutkar A, Bell EL, Zhang F, Langer R, Sharp PA. 2014. Global microRNA depletion suppresses tumor angiogenesis. Genes Dev 28:1054–1067. 10.1101/gad.239681.114. 24788094 PMC4035535

[35] Huang B, Zhao J, Lei Z, Shen S, Li D, Shen GX, Zhang GM, Feng ZH. 2009. miR-142-3p restricts cAMP production in CD4^+^CD25^−^ T cells and CD4^+^CD25^+^ TREG cells by targeting AC9 mRNA. EMBO Rep 10:180–185. 10.1038/embor.2008.224. 19098714 PMC2637310

[36] Wang X, Guo Y, Wang C, Yu H, Yu X, Yu H. 2016. MicroRNA-142-3p inhibits chondrocyte apoptosis and inflammation in osteoarthritis by targeting HMGB1. Inflammation 39:1718–1728. 10.1007/s10753-016-0406-3. 27447821

[37] Prabahar A, Natarajan J. 2017. ImmunemiR—a database of prioritized immune miRNA disease associations and its interactome. Microrna 6:71–78. 10.2174/2211536606666170117112322. 28124611

[38] Nahid MA, Pauley KM, Satoh M, Chan EK. 2009. miR-146a is critical for endotoxin-induced tolerance: implication in innate immunity. J Biol Chem 284:34590–34599. 10.1074/jbc.M109.056317. 19840932 PMC2787321

[39] Matsuzawa A, Saegusa K, Noguchi T, Sadamitsu C, Nishitoh H, Nagai S, Koyasu S, Matsumoto K, Takeda K, Ichijo H. 2005. ROS-dependent activation of the TRAF6-ASK1-p38 pathway is selectively required for TLR4-mediated innate immunity. Nat Immunol 6:587–592. 10.1038/ni1200. 15864310

[40] Wee ZN, Yatim SMJM, Kohlbauer VK, Feng M, Goh JY, Bao Y, Yi B, Lee PL, Zhang S, Wang PP, Lim E, Tam WL, Cai Y, Ditzel HJ, Hoon DSB, Tan EY, Yu Q. 2015. IRAK1 is a therapeutic target that drives breast cancer metastasis and resistance to paclitaxel. Nat Commun 6:8746. 10.1038/ncomms9746. 26503059 PMC4640083

[41] Shi Y, Tu Z, Tang D, Zhang H, Liu M, Wang K, Calderwood SK, Xiao X. 2006. The inhibition of LPS-induced production of inflammatory cytokines by HSP70 involves inactivation of the NF-kappaB pathway but not the MAPK pathways. Shock 26:277–284. 10.1097/01.shk.0000223134.17877.ad. 16912653

[42] Dokladny K, Lobb R, Wharton W, Ma TY, Moseley PL. 2010. LPS-induced cytokine levels are repressed by elevated expression of HSP70 in rats: possible role of NF-kappaB. Cell Stress Chaperones 15:153–163. 10.1007/s12192-009-0129-6. 19551494 PMC2866987

[43] Ananieva O, Darragh J, Johansen C, Carr JM, McIlrath J, Park JM, Wingate A, Monk CE, Toth R, Santos SG, Iversen L, Arthur JS. 2008. The kinases MSK1 and MSK2 act as negative regulators of Toll-like receptor signaling. Nat Immunol 9:1028–1036. 10.1038/ni.1644. 18690222

[44] Lowenstein CJ, Alley EW, Raval P, Snowman AM, Snyder SH, Russell SW, Murphy WJ. 1993. Macrophage nitric oxide synthase gene: two upstream regions mediate induction by interferon gamma and lipopolysaccharide. Proc Natl Acad Sci USA 90:9730–9734. 10.1073/pnas.90.20.9730. 7692452 PMC47644

[45] Xaus J, Comalada M, Valledor AF, Lloberas J, Lopez-Soriano F, Argiles JM, Bogdan C, Celada A. 2000. LPS induces apoptosis in macrophages mostly through the autocrine production of TNF-alpha. Blood 95:3823–3831. 10.1182/blood.V95.12.3823. 10845916

[46] Boulares AH, Yakovlev AG, Ivanova V, Stoica BA, Wang G, Iyer S, Smulson M. 1999. Role of poly(ADP-ribose) polymerase (PARP) cleavage in apoptosis. Caspase 3-resistant PARP mutant increases rates of apoptosis in transfected cells. J Biol Chem 274:22932–22940. 10.1074/jbc.274.33.22932. 10438458

[47] Jablonski KA, Gaudet AD, Amici SA, Popovich PG, Guerau-de-Arellano M. 2016. Control of the inflammatory macrophage transcriptional signature by miR-155. PLoS One 11:e0159724. 10.1371/journal.pone.0159724. 27447824 PMC4957803

[48] Lu L, McCurdy S, Huang S, Zhu X, Peplowska K, Tiirikainen M, Boisvert WA, Garmire LX. 2016. Time series miRNA-mRNA integrated analysis reveals critical miRNAs and targets in macrophage polarization. Sci Rep 6:37446. 10.1038/srep37446. 27981970 PMC5159803

[49] Gerrits A, Walasek MA, Olthof S, Weersing E, Ritsema M, Zwart E, van Os R, Bystrykh LV, de Haan G. 2012. Genetic screen identifies microRNA cluster 99b/let-7e/125a as a regulator of primitive hematopoietic cells. Blood 119:377–387. 10.1182/blood-2011-01-331686. 22123844

[50] Ajuyah P, Hill M, Ahadi A, Lu J, Hutvagner G, Tran N. 2019. MicroRNA (miRNA)-to-miRNA regulation of programmed cell death 4 (PDCD4). Mol Cell Biol 39:e00086-19. 10.1128/MCB.00086-19. 31235478 PMC6712940

[51] Mann M, Mehta A, Zhao JL, Lee K, Marinov GK, Garcia-Flores Y, Lu LF, Rudensky AY, Baltimore D. 2017. An NF-kappaB-microRNA regulatory network tunes macrophage inflammatory responses. Nat Commun 8:851. 10.1038/s41467-017-00972-z. 29021573 PMC5636846

[52] Huffaker TB, Hu R, Runtsch MC, Bake E, Chen X, Zhao J, Round JL, Baltimore D, O'Connell RM. 2012. Epistasis between microRNAs 155 and 146a during T cell-mediated antitumor immunity. Cell Rep 2:1697–1709. 10.1016/j.celrep.2012.10.025. 23200854 PMC3628775

[53] Roux PP, Blenis J. 2004. ERK and p38 MAPK-activated protein kinases: a family of protein kinases with diverse biological functions. Microbiol Mol Biol Rev 68:320–344. 10.1128/MMBR.68.2.320-344.2004. 15187187 PMC419926

[54] Chi H, Barry SP, Roth RJ, Wu JJ, Jones EA, Bennett AM, Flavell RA. 2006. Dynamic regulation of pro- and anti-inflammatory cytokines by MAPK phosphatase 1 (MKP-1) in innate immune responses. Proc Natl Acad Sci USA 103:2274–2279. 10.1073/pnas.0510965103. 16461893 PMC1413743

[55] Chuang SM, Wang IC, Yang JL. 2000. Roles of JNK, p38 and ERK mitogen-activated protein kinases in the growth inhibition and apoptosis induced by cadmium. Carcinogenesis 21:1423–1432. 10.1093/carcin/21.7.1423. 10874022

[56] Chen CC, Wang JK. 1999. p38 but not p44/42 mitogen-activated protein kinase is required for nitric oxide synthase induction mediated by lipopolysaccharide in RAW264.7 macrophages. Mol Pharmacol 55:481–488. 10051531

[57] Chan ED, Riches DW. 2001. IFN-gamma + LPS induction of iNOS is modulated by ERK, JNK/SAPK, and p38(mapk) in a mouse macrophage cell line. Am J Physiol Cell Physiol 280:C441–C450. 10.1152/ajpcell.2001.280.3.C441. 11171562

[58] Tili E, Michaille JJ, Cimino A, Costinean S, Dumitru CD, Adair B, Fabbri M, Alder H, Liu CG, Calin GA, Croce CM. 2007. Modulation of miR-155 and miR-125b levels following lipopolysaccharide/TNF-alpha stimulation and their possible roles in regulating the response to endotoxin shock. J Immunol 179:5082–5089. 10.4049/jimmunol.179.8.5082. 17911593

[59] Canfran-Duque A, Rotllan N, Zhang X, Fernandez-Fuertes M, Ramirez-Hidalgo C, Araldi E, Daimiel L, Busto R, Fernandez-Hernando C, Suarez Y. 2017. Macrophage deficiency of miR-21 promotes apoptosis, plaque necrosis, and vascular inflammation during atherogenesis. EMBO Mol Med 9:1244–1262. 10.15252/emmm.201607492. 28674080 PMC5582411

[60] Kim SW, Ramasamy K, Bouamar H, Lin AP, Jiang D, Aguiar RC. 2012. MicroRNAs miR-125a and miR-125b constitutively activate the NF-kappaB pathway by targeting the tumor necrosis factor alpha-induced protein 3 (TNFAIP3, A20). Proc Natl Acad Sci USA 109:7865–7870. 10.1073/pnas.1200081109. 22550173 PMC3356650

[61] Rajaram MV, Ni B, Morris JD, Brooks MN, Carlson TK, Bakthavachalu B, Schoenberg DR, Torrelles JB, Schlesinger LS. 2011. Mycobacterium tuberculosis lipomannan blocks TNF biosynthesis by regulating macrophage MAPK-activated protein kinase 2 (MK2) and microRNA miR-125b. Proc Natl Acad Sci USA 108:17408–17413. 10.1073/pnas.1112660108. 21969554 PMC3198317

[62] Meister G, Landthaler M, Patkaniowska A, Dorsett Y, Teng G, Tuschl T. 2004. Human Argonaute2 mediates RNA cleavage targeted by miRNAs and siRNAs. Mol Cell 15:185–197. 10.1016/j.molcel.2004.07.007. 15260970

[63] Barman B, Bhattacharyya SN. 2015. mRNA targeting to endoplasmic reticulum precedes Ago protein interaction and microRNA (miRNA)-mediated translation repression in mammalian cells. J Biol Chem 290:24650–24656. 10.1074/jbc.C115.661868. 26304123 PMC4598978

[64] Kundu P, Fabian MR, Sonenberg N, Bhattacharyya SN, Filipowicz W. 2012. HuR protein attenuates miRNA-mediated repression by promoting miRISC dissociation from the target RNA. Nucleic Acids Res 40:5088–5100. 10.1093/nar/gks148. 22362743 PMC3367187

[65] Bhattacharyya SN, Habermacher R, Martine U, Closs EI, Filipowicz W. 2006. Relief of microRNA-mediated translational repression in human cells subjected to stress. Cell 125:1111–1124. 10.1016/j.cell.2006.04.031. 16777601

[66] Ghosh K, Tang M, Kumari N, Nandy A, Basu S, Mall DP, Rai K, Biswas D. 2018. Positive regulation of transcription by human ZMYND8 through its association with P-TEFb complex. Cell Rep 24:2141–2154.e2146. 10.1016/j.celrep.2018.07.064. 30134174 PMC6152903

[67] Green LC, Wagner DA, Glogowski J, Skipper PL, Wishnok JS, Tannenbaum SR. 1982. Analysis of nitrate, nitrite, and [15N]nitrate in biological fluids. Anal Biochem 126:131–138. 10.1016/0003-2697(82)90118-X. 7181105

[68] Ghose AC, Mookerjee A, Sengupta K, Ghosh AK, Dasgupta S, Ray PK. 1999. Therapeutic and prophylactic uses of protein A in the control of Leishmania donovani infection in experimental animals. Immunol Lett 65:175–181. 10.1016/s0165-2478(98)00102-3. 10065740

[69] Simmonds RE. 2019. Transient up-regulation of miR-155-3p by lipopolysaccharide in primary human monocyte-derived macrophages results in RISC incorporation but does not alter TNF expression. Wellcome Open Res 4:43. 10.12688/wellcomeopenres.15065.2. 31641696 PMC6790912

[70] Vlachos IS, Paraskevopoulou MD, Karagkouni D, Georgakilas G, Vergoulis T, Kanellos I, Anastasopoulos IL, Maniou S, Karathanou K, Kalfakakou D, Fevgas A, Dalamagas T, Hatzigeorgiou AG. 2015. DIANA-TarBase v7.0: indexing more than half a million experimentally supported miRNA:mRNA interactions. Nucleic Acids Res 43:D153–D159. 10.1093/nar/gku1215. 25416803 PMC4383989

[71] Chou CH, Chang NW, Shrestha S, Hsu SD, Lin YL, Lee WH, Yang CD, Hong HC, Wei TY, Tu SJ, Tsai TR, Ho SY, Jian TY, Wu HY, Chen PR, Lin NC, Huang HT, Yang TL, Pai CY, Tai CS, Chen WL, Huang CY, Liu CC, Weng SL, Liao KW, Hsu WL, Huang HD. 2016. miRTarBase 2016: updates to the experimentally validated miRNA-target interactions database. Nucleic Acids Res 44:D239–D247. 10.1093/nar/gkv1258. 26590260 PMC4702890

[72] Schroder K, Irvine KM, Taylor MS, Bokil NJ, Le Cao KA, Masterman KA, Labzin LI, Semple CA, Kapetanovic R, Fairbairn L, Akalin A, Faulkner GJ, Baillie JK, Gongora M, Daub CO, Kawaji H, McLachlan GJ, Goldman N, Grimmond SM, Carninci P, Suzuki H, Hayashizaki Y, Lenhard B, Hume DA, Sweet MJ. 2012. Conservation and divergence in Toll-like receptor 4-regulated gene expression in primary human versus mouse macrophages. Proc Natl Acad Sci USA 109:E944–E953. 10.1073/pnas.1110156109. 22451944 PMC3341041

[73] Regan T, Gill AC, Clohisey SM, Barnett MW, Pariante CM, Harrison NA, Hume DA, Bullmore ET, Freeman TC, MRC Immunopsychiatry Consortium. 2018. Effects of anti-inflammatory drugs on the expression of tryptophan-metabolism genes by human macrophages. J Leukoc Biol 103:681–692. 10.1002/JLB.3A0617-261R. 29377288 PMC5918594

[74] Yu G, Wang LG, Han Y, He QY. 2012. clusterProfiler: an R package for comparing biological themes among gene clusters. OMICS 16:284–287. 10.1089/omi.2011.0118. 22455463 PMC3339379

[75] Geng S, Chen K, Yuan R, Peng L, Maitra U, Diao N, Chen C, Zhang Y, Hu Y, Qi CF, Pierce S, Ling W, Xiong H, Li L. 2016. The persistence of low-grade inflammatory monocytes contributes to aggravated atherosclerosis. Nat Commun 7:13436. 10.1038/ncomms13436. 27824038 PMC5105176

[76] Lin Y, Duan Z, Xu F, Zhang J, Shulgina MV, Li F. 2017. Construction and analysis of the transcription factor-microRNA co-regulatory network response to Mycobacterium tuberculosis: a view from the blood. Am J Transl Res 9:1962–1976. 28469803 PMC5411946

[77] Edgar R, Domrachev M, Lash AE. 2002. Gene expression omnibus: NCBI gene expression and hybridization array data repository. Nucleic Acids Res 30(1):207–10. 11752295 10.1093/nar/30.1.207 PMC99122

[78] Barrett T, Wilhite SE, Ledoux P, Evangelista C, Kim IF, Tomashevsky M, Marshall KA, Phillippy KH, Sherman PM, Holko M, Yefanov A, Lee H, Zhang N, Robertson CL, Serova N, Davis S, Soboleva A. 2012. NCBI GEO: archive for functional genomics data sets--update. Nucleic Acids Res 41:D991–5. 23193258 10.1093/nar/gks1193 PMC3531084

[79] Kanehisa M, Goto S. 2000. KEGG: kyoto encyclopedia of genes and genomes. Nucleic Acids Res 28(1):27–30. 10.1093/nar/28.1.27. 10592173 PMC102409

[80] R Core Team. 2018. R: a language and environment for statistical computing. https://www.R-project.org.

[81] Ritchie ME, Phipson B, Wu D, Hu Y, Law CW, Shi W, Smyth GK. 2015. limma powers differential expression analyses for RNA-sequencing and microarray studies. Nucleic Acids Res 43(7):e47. 10.1093/nar/gkv007. 25605792 PMC4402510

