# Supplementary material for: Target-Dependent Coordinated Biogenesis of Secondary MicroRNAs by miR-146a Balances Macrophage Activation Processes
Source: Mol Cell Biol. 2022 Mar 21;42(4):e00452-21. doi: 10.1128/mcb.00452-21 (PMC9022539; doi:10.1128/mcb.00452-21)
Supplement: Supplemental file 7 — Captions to Tables S1 to S6. Download mcb.00452-21-s0007.pdf, PDF file, 0.1 MB [file mcb.00452-21-s0007.pdf]

Table S1 List of possible CB regulator-target relationships identified considering mmu-miR-146a-5p as CB regulator, which modulate immune response in murine macrophages. The fold change status of differentially expressed mRNA inmurine macrophages exposed to LPS (10ng/ml) for 24 hours (GSE19490) have been included for down-regulated 'Gene A' and 'Gene-B'.

Table S2 List of probable CB regulator-target relationships identified considering hsa-miR-146a-5p as a regulator, in human macrophages upon LPS exposure. 'GeneA' and 'Gene-B' that were found tobe down-regulated based on the fold change status of differentially expressed mRNA inmacrophages exposed to LPS (10ng/ml) for 24 hours (GSE85333) are shown.

Table S3 miR-146a-5p influences immune response in macrophages by co-ordinately regulating the expression of other miRNA (secondary effector).

A miR-146a-5p co-ordinate biogenesis regulatory network in primary human monocyte-derived macrophages stimulated with 100ng/ml LPS (Lipopolysaccharide) for 8 hours.

B miR-146a-5p co-ordinate biogenesis regulatory network during *Mycobacterium tuberculosis* (*Mtb*) infection.

Table S4 Differentially expressed miRNA upon inducible miR-146a-5p over-expression in murine macrophage cells (RAW264.7).

Table S5 Probable CB regulator-target relationships.

A Considering mmu-miR-155-5p as regulator in murine macrophages (miR-155 knock-out) responding to LPS.

B Considering mmu-miR-155-5p as regulator during macrophage polarization.

C Considering mmu-miR-125a-3p as CB-regulator and transcriptional profile during macrophage polarization.

D Considering mmu-miR-125a-3p as CB-regulator with transcriptional profile in hematopoietic stem cell differentiation.

Table S6 All possible CB regulator-target relationships identified by considering mmu-miR-146a-5p as CB regulator (without conditional or expression-based selection).
